# Targeting WD repeat domain 5 enhances chemosensitivity and inhibits proliferation and programmed death-ligand 1 expression in bladder cancer

**DOI:** 10.1186/s13046-021-01989-5

**Published:** 2021-06-21

**Authors:** Jingtong Zhang, Qianghua Zhou, Keji Xie, Liang Cheng, Shengmeng Peng, Ruihui Xie, Lixuan Liu, Yangjie Zhang, Wen Dong, Jinli Han, Ming Huang, Yuelong Chen, Tianxin Lin, Jian Huang, Xu Chen

**Affiliations:** 1 Department of Urology, Sun Yat-sen Memorial Hospital, Sun Yat-sen University, 107th Yanjiangxi Road, Guangzhou, China; 2 Guangdong Provincial Key Laboratory of Malignant Tumor Epigenetics and Gene Regulation, Sun Yat-sen Memorial Hospital, Sun Yat-sen University, Guangzhou, China; 3 Guangdong Provincial Clinical Research Center for Urological Diseases, Guangzhou, China; 4 Department of Urology, Guangzhou First People’s Hospital, Guangzhou, China; 5 Department of Endocrinology, Sun Yat-sen Memorial Hospital, Sun Yat-sen University, Guangzhou, China; 6 Department of Urology, The 1st Affiliated Hospital of Kunming Medical University, Kunming, China; 7 Department of Urology, The Affiliated Kashi Hospital, Sun Yat-sen University, Kashi, China

**Keywords:** WDR5 inhibitor, OICR-9429, Bladder cancer, PD-L1, Target therapy, Chemosensitivity, Metastasis

## Abstract

**Background:**

Chemotherapy and/or immunotherapy are first-line treatments for advanced muscle-invasive bladder cancer (BCa), but the unsatisfactory objective response rate to these treatments yields poor 5-year patient survival. Discovery of therapeutic targets essential for BCa maintenance is critical to improve therapy response in clinic. This study evaluated the role of targeting WD repeat domain 5 (WDR5) with the small molecule compound OICR-9429 and whether it could be used to treat bladder cancer.

**Methods:**

We analysed the expression and clinical prognosis of WDR5 in a TCGA cohort. The pharmacological role of OICR-9429 was further investigated in vitro and in vivo. RNA sequencing, western blot, and chromatin immunoprecipitation (ChIP) were utilized to explored the mechanism underlying OICR-9429-induced WDR5 inhibition.

**Results:**

First, we found that WDR5 expression was upregulated in BCa and was associated with histologic grade, metastasis status, histologic subtype, and molecular subtype. High WDR5 expression level was also correlated with shorter overall survival (OS) in BCa. The WDR5 inhibitor OICR-9429 reduced cell viability by decreasing H3K4me3 levels but not WDR5 levels in T24, UM-UC-3, and TCCSUP BCa cells. OICR-9429 suppressed the proliferation of BCa cells by blocking the G1/S phase transition. Next, OICR-9429 enhanced apoptosis and chemosensitivity to cisplatin in BCa cells. In addition, OICR-9429 independently inhibited the motility and metastatic behaviour of BCa cells. In vivo experiments further revealed that OICR-9429 suppressed tumour growth, enhanced chemosensitivity, and reduced the toxicity of cisplatin in BCa. Notably, WDR5 was positively correlated with programmed death-ligand 1 (PD-L1) expression, and OICR-9429 suppressed immune evasion by blocking PD-L1 induced by IFN-γ. Mechanistically, some cell cycle-, antiapoptosis-, DNA repair-, metastasis-, and immune evasion-related genes, including BIRC5, XRCC2, CCNB1, CCNE2, PLK1, AURKA, FOXM1, and PD-L1 were identified to be directly regulated by OICR-9429 in a H3K4me3-dependent manner.

**Conclusions:**

Our novel finding is that the WDR5 inhibitor, OICR-9429, suppressed proliferation, metastasis and PD-L1-based immune evasion while enhancing apoptosis and chemosensitivity to cisplatin in BCa by blocking the WDR5-MLL complex mediating H3K4me3 in target genes. Hence, our findings offer insight into a multipotential anticancer compound, OICR-9429, which enhances the antitumour effect of cisplatin or immunotherapy in BCa.

**Supplementary Information:**

The online version contains supplementary material available at 10.1186/s13046-021-01989-5.

## Background

Worldwide, bladder cancer (BCa) is the most common malignancy of the urinary system with more than 573,278 new cases and 212,536 deaths worldwide in 2020 [1, 2]. Muscle-invasive BCa (MIBC) represents 25–40% of all BCa and is a life-threatening disease that can spread from the bladder to other organs, leading to almost 100% of deaths from this disease [3, 4]. Cisplatin-based chemotherapy is the standard treatment for MIBC, but it provides a median survival of only approximately 14 months for metastatic BCa. Little improvement in survival has been attained over the last few decades [5]. Novel immunotherapy, represented by monoclonal antibodies against programmed cell death-1 (PD-1) and its ligand, PD-L1, has been recently approved by the US Food and Drug Administration (FDA) for treating of BCa. However, the objective response rate of these checkpoint inhibitors in BCa is only 17.8–24% [6]. Therefore, it is urgent to develop novel therapeutics for MIBC, either as single agents or in combination with standard chemotherapy regimens.

Epigenetic regulation, which includes DNA methylation and histone modification, plays a vital role in gene expression [7, 8]. WDR5, a histone H3 lysine 4 (H3K4) presenter, together with H3K4 methyltransferases MLL1-MLL4 and other protein subunits, DPY30, ASH2L, and RBBP5, forms protein complexes and plays a vital role in histone methylation, chromatin remodelling, transcriptional activation of target genes, and normal and disease biology [9–11]. Recent studies have demonstrated that WDR5 is upregulated in a variety of cancers, including leukaemia, bladder, prostate, and colon cancers, and participates in tumorigenesis and progression [12–15]. Our previous study showed that WDR5 played an oncogene role in proliferation, self-renewing, and cisplatin chemoresistance of BCa [13]. Therefore, WDR5 may be a promising target in the treatment of BCa.

Accumulating evidence indicates that epigenetic proteins are druggable targets, and FDA has already approved two classes of epigenetic drugs for cancer therapy [16]. WDR5 inhibitors, which have been developed to block MLL-WDR5 protein-protein interactions and subsequent transcription of oncogenic gene, are emerging as novel anticancer agents in preclinical development [11]. OICR-9429, a high affinity small molecule compound, competitively blocks WDR5 interaction with MLL protein via binding the central peptide-binding pocket of WDR5 [17]. OICR-9429 has anticancer efficacy against non-MLL-rearranged leukaemia and colon, pancreatic, and prostate cancer by potently suppressing histone H3K4 trimethylation [14, 15, 18]. However, the role of targeting WDR5 with the small molecule compound OICR-9429 and whether it could be used in the treatment of BCa remain to be further investigated.

In this study, we identified that the WDR5 inhibitor, OICR-9429, reduced cell viability by decreasing H3K4me3 levels but not WDR5 in T24, UM-UC-3, and TCCSUP BCa cells. Furthermore, OICR-9429 suppressed cell proliferation and metastatic behaviour, but promoted cell apoptosis and cisplatin chemosensitivity in BCa cells*.* Additionally, WDR5 expression had positive correlation with PD-L1, and OICR-9429 reduced PD-L1 expression induced by IFN-γ in BCa. Mechanistically, through RNA sequencing, western blot, and chromatin immunoprecipitation (ChIP), we identified some cell cycle-, metastasis- and anti-apoptosis-related genes of OICR-9429. In summary, targeting WDR5 with OICR-9429 is a multipotency anticancer therapy that enhances the antitumour effect of cisplatin or immunotherapy in BCa.

## Materials and methods

### TCGA data mining

Patient clinical profiles in the TCGA cohort are available at the website of The Cancer Genome Atlas Program (https://cancergenome.nih.gov) [19]. A total number of 408 patients in the TCGA bladder cancer cohort was used for the analysis. Patients with no available clinical data were removed in clinical characters analysis. For the Kaplan–Meier survival, univariate, and multivariate analyses, 345 cases with complete follow-up information were included.

### Cell culture

Three BCa cell lines were used in this study, including T24, UM-UC-3, and TCCSUP (ATCC, Manassas, USA). T24 cells were cultured in Roswell Park Memorial Institute (RPMI)-1640 (Gibco, Shanghai, China), whereas UM-UC-3 and TCCSUP cells were cultured in Dulbecco’s modified Eagle’s medium (DMEM) (Gibco, Shanghai, China). All media were supplemented with 10% fetal bovine serum (FBS; Biological Industries, 04–001-1ACS) and 1% penicillin/streptomycin (Gibco, USA). The WDR5 inhibitor (OICR-9429) was purchased from Selleck (Houston, TX, USA) and dissolved in DMSO. Cells were grown in a 37 °C, 5% CO_2_ cell incubator with humidified atmosphere. The cells used in this study were excluded from mycoplasma contamination. Short tandem repeat (STR) authentication of the bladder cancer cell lines T24, UM-UC-3, and TCCSUP was conducted to prove that there was no misidentification or contamination with other cells. STR authentications were conducted by IGE Biotechnology LTD, Guangzhou, China.

### Cell proliferation assay

Cell viability was detected by the colony formation assay and the 3-(4,5-dimethylthiazol-2-yl)-2,5-diphenyltetrazolium bromide (MTT) assay. Cell cycle analysis and ethynyl deoxyuridine (EdU) assay were performed to detect cell populations at different cell cycle phases. The above experiments were conducted as described in our previous publication [20].

### Cytotoxicity and chemosensitivity assay

The indicated cells were treated with 0, 10, 20, 30, 40, 50, 60, 70, 80, 90, 100, 125, 150, 200, 300, and 400 μM OICR-9429 for 48 h. The indicated cells were treated with 0, 0.5, 1, 1.5, 2, and 2.5 μg/ml cisplatin (Sigma) and IC_50_ and 2 IC_50_ OICR-9429 for 48 h. Cell viability was detected using the MTT assay. To calculate the half inhibitory concentration (IC_50_), data were fitted in GraphPad Prism 9 (GraphPad Software Inc., San Diego, CA, USA), and a dose-response curve was plotted using the equation log (inhibitor) vs. response-variable slope. This curve is also called a four-parameter dose-response curve: Y = Bottom + (Top-Bottom) / {1 + 10 ^ [(Log IC_50_-X) *HillSlope]}. We used 70 μM as the IC_50_ dose for T24 and UM-UC-3 cells and 120 μM as the IC_50_ dose for TCCSUP cells. As for synergistic effect, two BCa cell lines were treated with OICR-9429 and cisplatin individually or in combination at indicated concentrations for 48 h. Cell viabilities were measured and normalized to control group. For the combinational index (CI), CalcuSyn (Biosoft) was used to calculate CI at different drug concentration. CI < 1.0 demonstrates synergistic effect between two drugs.

### Apoptosis analysis

The apoptosis analysis, the caspase-3/7 assay, and the terminal deoxynucleotidyl transferase (TdT) dUTP nick end labelling (TUNEL) assay were conducted as previously described [21–23].

### Comet assay

The comet assay was performed using Trevigen CometAssay kit following the manufacturer’s instructions. CometScore 2.0 was used to calculate the olive tail movement. At least 50 cells were analysed and plotted.

### In vivo Xenograft mouse model and chemotherapy assay

Animal experiments were approved by the Institutional Animal Care and Use Committee, Sun Yat-sen University. Male BALB/c nude mice (4–5 weeks old) were obtained from the Experimental Animal Center, Sun Yat-sen University. All procedures involving animals were proceeded in specific pathogen-free (SPF) barrier facilities. For the xenograft mouse model, a total of 3 × 10^6^ UM-UC-3 cells were injected subcutaneously into the right side of the dorsum, and there were 6 nude mice in every treatment group. Five days after injection, tumour-bearing mice were randomly assigned into four groups that received control solvent, OICR-9429 (60 mg/kg), cisplatin (4 mg/kg), or a combination of small dose OICR-9429 (30 mg/kg) and cisplatin (2.5 mg/kg). The dosage of cisplatin was based on our previous research [21]. OICR-9429 was diluted in a combination solution of 5% DMSO + 40% PEG300 + 2% Tween 80 + 53% sterilized water, and cisplatin was diluted in sterilized PBS solution. OICR-9429 and chemotherapy were given every 2 days by intraperitoneal injection. After 16 days of treatment, the mice were euthanized, and tumours were surgically dissected. The tumour and organ specimens were fixed in 4% paraformaldehyde, embedded in paraffin, and then stained with H&E staining. The tumour volumes were calculated using the following formula: tumour volume (mm^3^) = [length (mm)] × [width (mm)]^2^ × 0.52.

### Immunohistochemistry (IHC) staining and scoring analyses

The immunohistochemistry (IHC) experiment and IHC score calculations were conducted as previously described [22]. Briefly, an anti-Ki67 antibody (1:100, Service Bio, Wuhan, China) was used to detect the Ki67 expression in paraformaldehyde-fixed, paraffin-embedded murine tumour tissue. Images were obtained by ECLIPSE Ti microscope system (Nikon, Japan).

### In vitro cell wound healing, migration, and invasion assay

Wound healing assay and Transwell assay were conducted as described in our previous study to detect cell migration and invasion [20, 24].

### Western blot

Western blots were conducted as previously described [25]. Primary antibodies specific to CDK1, CCNB1, CCNE2, BIRC5, AURKA, FOXM1, WDR5, H3K4me3 (1:1000, Cell Signalling Technology, Danvers, MA, USA), histone H3 (1:2000, Cell Signalling Technology, Danvers, MA, USA), XRCC2, MCM2, PLK1, and GAPDH (1:1000, Abcam) were used. The PVDF films were then incubated with secondary antibodies (anti-mouse or anti-rabbit, Promega, USA) and visualized using Immobilon enhanced chemiluminescence (Millipore). The full images of all Western blots are shown in Supplementary Fig. 9.

### RNA sequencing analysis

T24 and UM-UC-3 cells were treated with OICR-9429 (2 IC_50_) or DMSO for 48 h (*n* = 4). The total RNA was extracted from treated cells by using TRIzol (Invitrogen). Library construction and RNA-sequencing were conducted by Annoroad Gene Technology (Beijing, China). The libraries were sequenced on an Illumina NovaSeq 6000 (S4) platform, and 150-bp paired-end reads were generated. ENSEMBL database (http://www.ensembl.org/index.html) were downloaded and used as reference genomes. Bowtie2 v2.2.3 was used to build the genome index, and HISAT2 v2.1.0. was used to align clean data to the reference genome. Differential gene expression analysis was performed by DEGseq. Raw data in RNA-sequencing analysis were submitted to the Gene Expression Omnibus (GSE145158).

### RNA isolation and qRT-PCR

RNA isolation and qRT-PCR were performed as previously described [23]. Relative expression was calculated using the 2^-∆∆Ct^ method (Ct, cycle threshold). All specific primers used in qRT-PCR are listed in Supplementary Table 2.

### ChIP

ChIP was conducted using Pierce magnetic ChIP kit (Thermo Scientific, USA) according to the manufacturer’s instructions. Briefly, after treatment with OICR-9429 (2 IC_50_) or DMSO for 48 h, T24 and UM-UC-3 cells were then crosslinked with 1% formaldehyde, lysed with IP lysis buffer, digested with micrococcal nuclease, ultrasonicated, and incubated with anti-IgG, anti-H3K4me3, and anti-RNA polymerase-II antibodies respectively. After incubation with protein A/G magnetic beads and washing with wash buffers, chromatin fragments were harvested using the elution buffer. Finally, after decrosslinking and protein digestion, the resulting DNA fragments were purified and fold enrichment was examined using qPCR. Primers for ChIP-qPCR are listed in Supplementary Table 3.

### Illustration

Mechanism illustration was created with BioRender.com. A Venn diagram was created with Venny (version 2.1, https://bioinfogp.cnb.csic.es/tools/venny). A Gene Ontology analysis diagram was created with Metascape [26] (http://metascape.org).

### Statistical analyses

Data are presented as the mean ± SD from three independent experiments. Each cell line experiment was performed three times. Two-tailed Student’s t-tests and one-way analysis of variance (ANOVA), followed by Dunnett’s tests for multiple comparisons, were used to evaluate the data. Cumulative survival time was calculated using the Kaplan-Meier method and analysed using the log-rank test. The best point cut-off value was used to define the gene expression level (low versus high) for all survival analyses in the present study. A multivariate Cox proportional hazards model was used to estimate the adjusted hazard ratios and 95% confidence intervals (CIs), and to identify independent prognostic factors. The Kruskal-Wallis test was used in the comet assay. All statistical analyses were performed with SPSS 19.0. A difference was considered statistically significant at *p < 0.05 and **p < 0.01.

## Results

### WDR5 is upregulated in bladder cancer and correlates with poor prognosis

Our previous study revealed that WDR5 was upregulated in BCa tissue by immunohistochemistry (IHC), and WDR5 expression positively correlated with the poor overall survival of BCa patients at Sun Yat-sen Memorial Hospital (SYSMH) cohort [13].

**Fig. 1:**
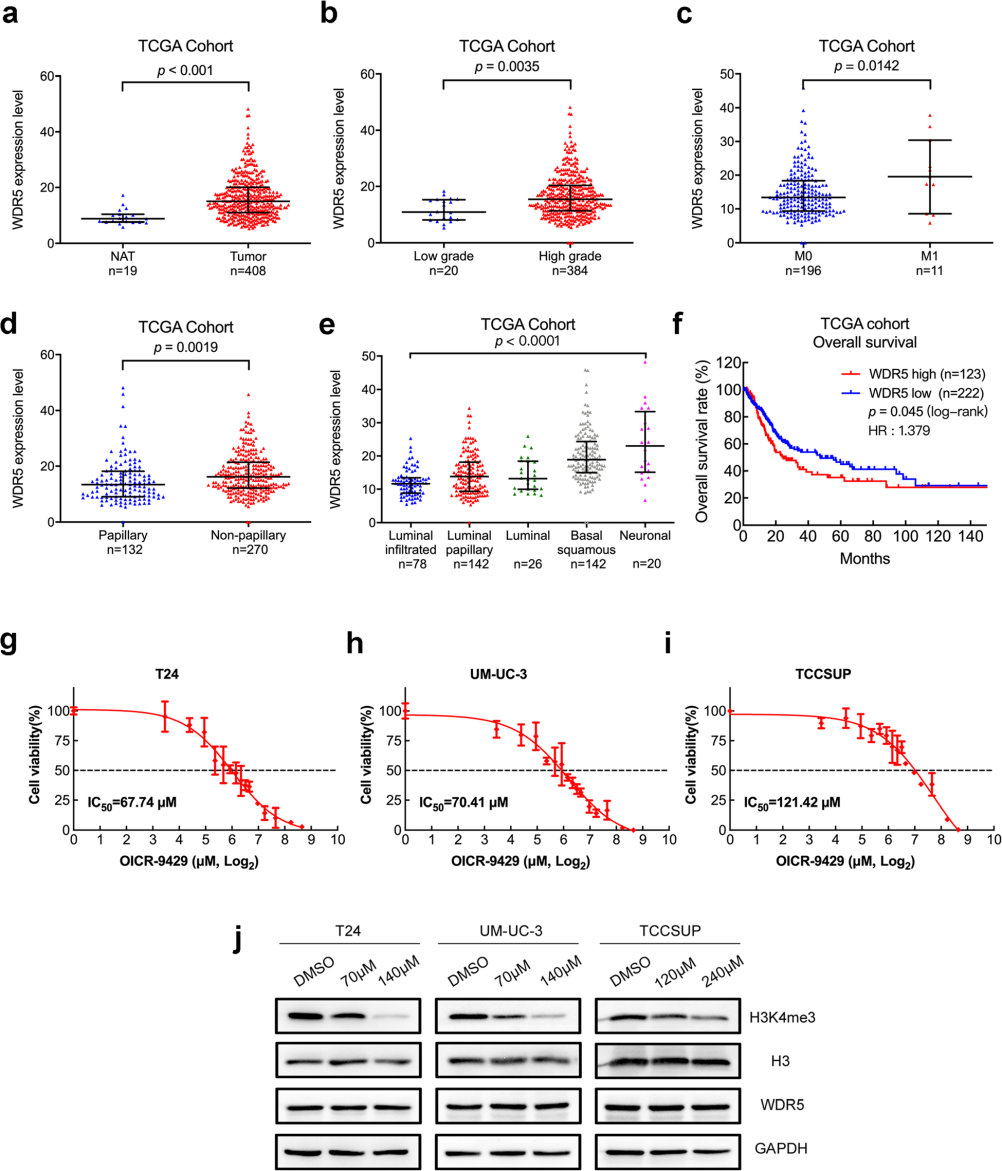
WDR5 associates with malignant features and is a potential therapy target in bladder cancer. **a.** WDR5 expression was detected in NAT and compared with BCa tissues in the TCGA cohort. **b-d.** WDR5 expression was detected between low grade and high grade (**b**), no distant metastasis (M0) and distant metastasis (M1) (**c**), papillary and non-papillary (**d**) BCa tumour tissues in TCGA cohort. **e.** WDR5 expression was detected in five molecular subtypes, including luminal infiltrated, luminal papillary, luminal, basal squamous and neuronal in TCGA cohort. **f.** Kaplan-Meier curves for OS in BCa patients with high vs. low expression of WDR5 in the TCGA cohort. **g-i.** Dose-reponse curves of T24 (**g**), UM-UC-3 (**h**), and TCCSUP (**i**) BCa cells treated with increasing concentrations of WDR5 inhibitor (OICR-9429) for 48 h. Cellular viability was determined by MTT assay, and the IC_50_ values were calculated based on nonlinear regression analysis. We used 70 μM as the IC_50_ dose for T24 and UM-UC-3 cells and 120 μM as the IC_50_ dose for TCCSUP cells. **j.** Western blots of H3K4me3, H3, and WDR5 protein levels in OICR-9429-treated BCa cells

To further investigate the role of WDR5 in BCa progression, we performed analyses of a 408-case cohort from The Cancer Genome Atlas (TCGA) database [19]. WDR5 was markedly overexpressed in tumour tissues compared with normal adjacent tissues (Fig. 1a). We next analysed WDR5 expression in association of clinical, pathological and molecular subtypes features in TCGA cohort. Intriguingly, WDR5 showed a higher expression in high grade, distant metastasis and non-papillary tumour tissues (Fig. 1b-d). In TCGA molecular subtypes, WDR5 expression was the lowest in luminal infiltrated tumour tissues, but gradually increased as malignancy progressed from luminal papillary, luminal, basal squamous, to neuronal tumour tissues. Moreover, WDR5 expression was markedly higher in basal squamous and neuronal BCa tissues than luminal infiltrated and papillary tissues, respectively (Fig. 1e). However, there was no significant difference among other features in TCGA cohort (SFig. 1). Furthermore, Kaplan–Meier survival analysis demonstrated that BCa patients with high WDR5 expression had markedly shorter overall survival (OS) in TCGA cohort (*p* = 0.045 and HR = 1.379, Fig. 1f). In addition, univariate analysis indicated that WDR5 expression was significantly associated with OS in TCGA cohort. Multivariate Cox regression analysis demonstrated that high WDR5 expression in BCa tissue was an independent prognostic factor for OS in TCGA cohort (Table S1). Collectively, these data show that WDR5 is overexpressed in BCa tumour tissues, and associated with advanced and malignant features, indicating that WDR5 may serve as a marker of poor prognosis in BCa.

### The cytotoxicity of OICR-9429 targeting WDR5 in bladder cancer cells

To investigate whether targeting WDR5 with OICR-9429 could be a promising therapy in BCa, we first detected the cytotoxicity of OICR-9429 on BCa cells by MTT assay. Interestingly, OICR-9429 obviously inhibited cell viability in a dose-dependent manner among the 3 BCa cell lines after treatment for 48 h (Fig. 1g, h, and I). T24 and UM-UC-3 showed high sensitivity, and the IC_50_ was 68 μM and 70 μM, whereas TCCSUP showed a low sensitivity to OICR-9429, and the IC_50_ was 121 μM. Hence, we used 70 μM as the IC_50_ dose for T24 and UM-UC-3 cells and 120 μM as the IC_50_ dose for TCCSUP cells. Then, we selected 2 concentrations (IC_50_, 2 IC_50_) of OICR-9429 to treat BCa cells for 48 h. Western blots showed significant downregulation of H3K4me3 in treated cells compared with the negative control at 48 h but not WDR5 or total H3 (Fig. 1j). Collectively, we verified that OICR-9429 reduced BCa cell viability by decreasing WDR5-mediated H3K4me3.

### OICR-9429 inhibits bladder cancer cell proliferation by regulating the cell cycle

**Fig. 2:**
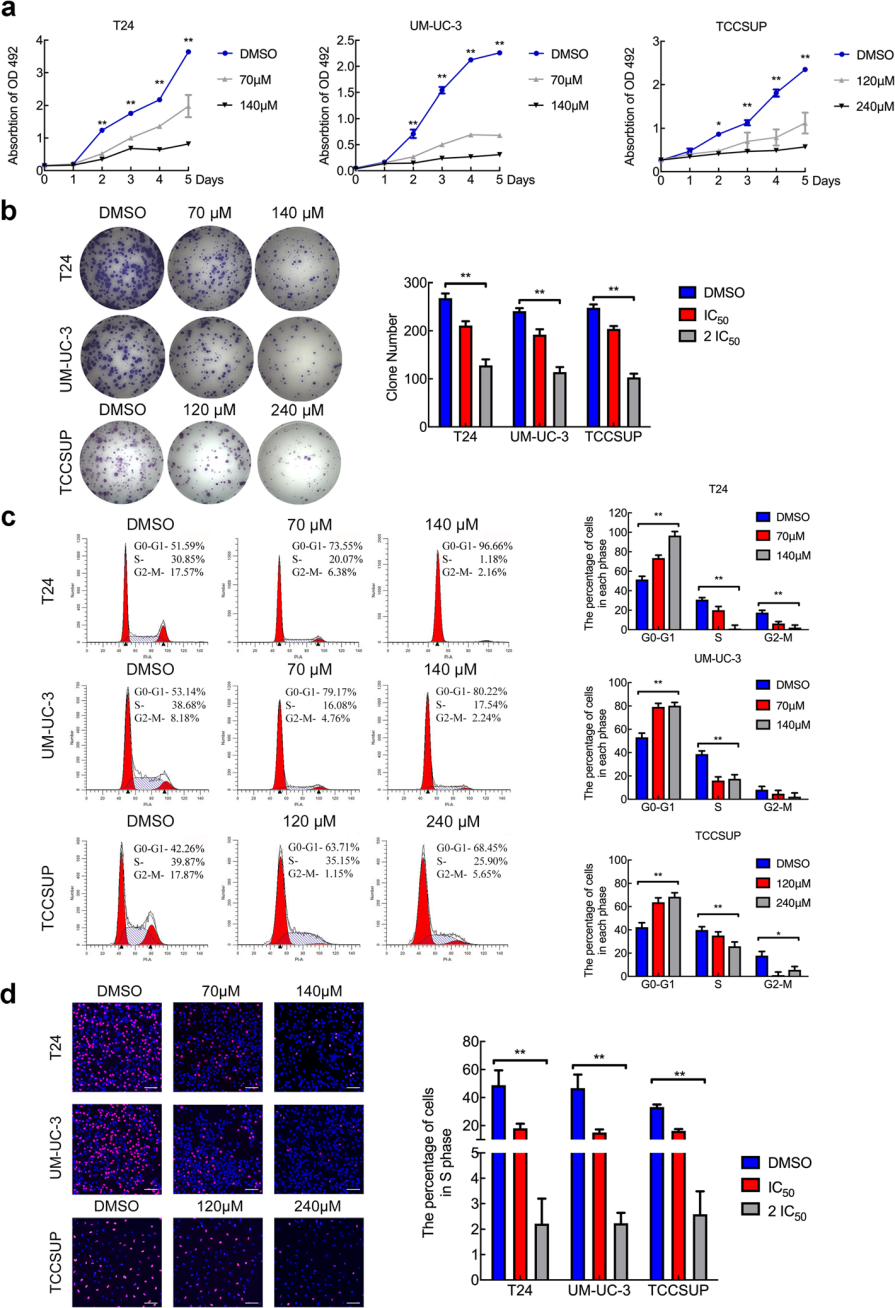
OICR-9429 inhibits bladder cancer cell proliferation by regulating the cell cycle. **a.** Cell viability was evaluated in T24, UM-UC-3, and TCCSUP BCa cells treated with OICR-9429 or DMSO for 5 days. **b.** The images and quantification of colony formation assays in three BCa cells treated with two OICR-9429 doses or DMSO. **c.** Representative images and quantification of the cell cycle in three BCa cells treated with OICR-9429 or DMSO for 48 h. The percentages (%) of cell populations at different stages of the cell cycle are listed in the panels. **d.** EdU assay measurement of the cell population in S phase and a histogram analysis of EdU-positive cell counts are shown. Blue indicates the nucleus; red indicates S-phase cells. Scale bars, 50 μm (white). *p < 0.05 and **p < 0.01

To detect whether OICR-9429 plays a role in cell proliferation, we investigated the function of OICR-9429 on BCa cells using MTT and colony formation assays. As shown in Fig. 2a, three BCa cells treated with OICR-9429 had a lower proliferation rate than the control cells, which was especially significant at high concentrations and 3 days later. Similarly, OICR-9429 remarkably reduced the number of colonies formed by the three BCa cell lines in a dose-dependent manner (Fig. 2b). To characterize whether OICR-9429 is involved in the regulation of the cell cycle, we conducted flow cytometry and EdU assays. Interestingly, after treatment with OICR-9429, the cell population in the G0/G1 phase of three BCa cells were dramatically increased, whereas cell population in the S and G2/M phases were reduced (Fig. 2c). Consistently, the EdU assay also revealed that OICR-9429 observably decreased the proportion of cells in S phase (Fig. 2d, SFig. 2). Taken together, these results indicated that WDR5 inhibition by OICR-9429 suppressed the proliferation of BCa cells by regulating the G1/S phase transition.

### OICR-9429 increases apoptosis and chemosensitivity of bladder cancer cells

**Fig. 3:**
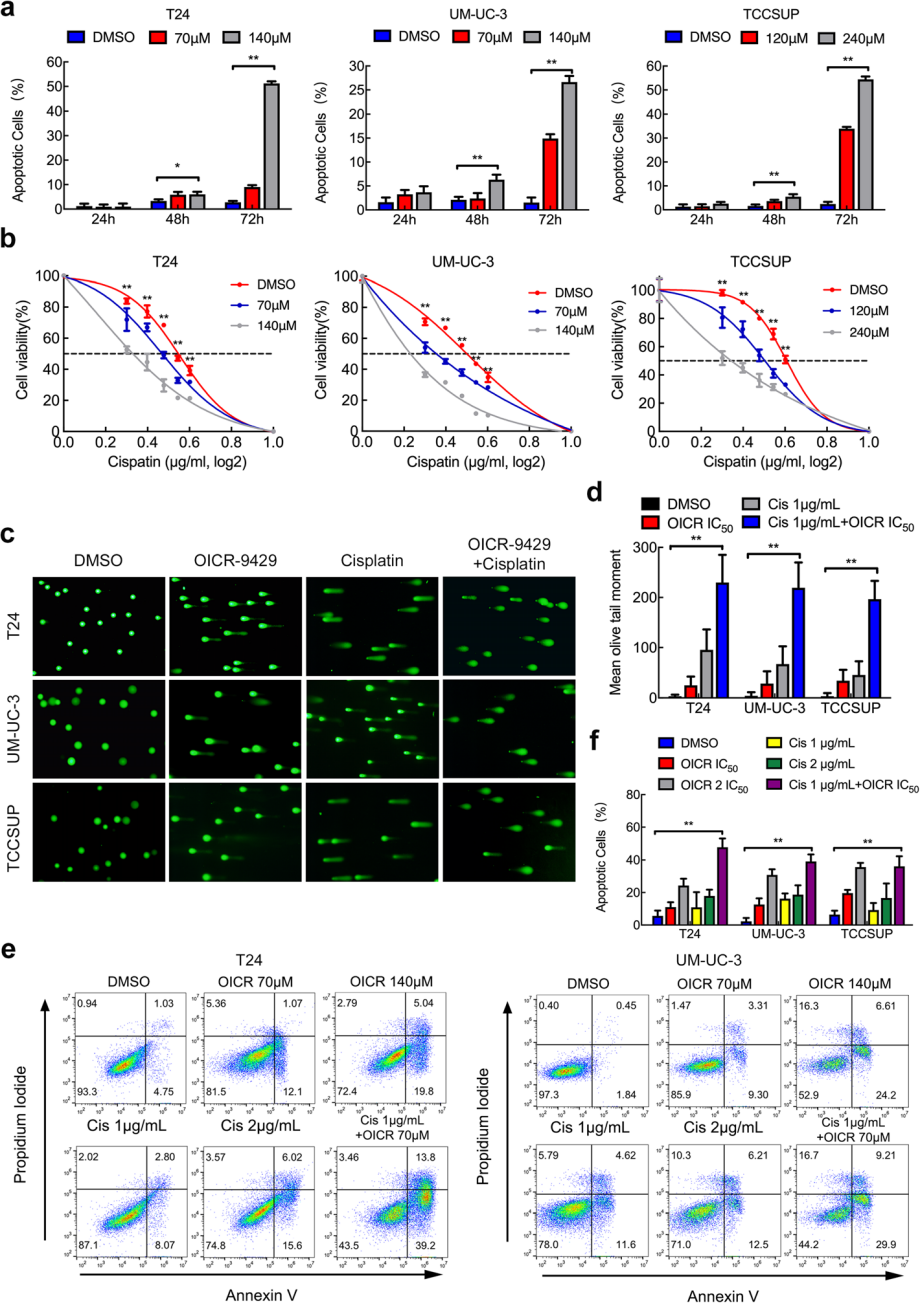
OICR-9429 increases apoptosis and chemosensitivity of bladder cancer cells. **a.** Apoptosis analysis of three BCa cell lines treated with two OICR-9429 doses or DMSO for 24, 48, and 72 h. The histogram shows the percentage (%) of apoptotic cells. **b.** Three BCa cells were treated with various concentrations of cisplatin combined with three concentrations of OICR-9429 (0, IC_50_, 2 IC_50_) for 72 h. Cell viability was determined by MTT assay. **c. d.** The comet assay (**c**) and histogram (**d**) analysis of three BCa cells treated with 2 IC_50_ OICR-9429, cisplatin (2 μg/mL), and a combined treatment with IC_50_ OICR-9429 and low-dose cisplatin (1 μg/mL) for 48 h. **e.** The apoptosis analysis of two BCa cells treated with 2 concentrations of OICR-9429 (IC_50_, 2 IC_50_), 2 concentrations of cisplatin (1, 2 μg/mL), and a combined treatment with IC_50_ OICR-9429 and low-dose cisplatin (1 μg/mL) for 72 h. **f.** The histogram showed the percentage (%) of apoptotic cells. *p < 0.05 and **p < 0.01

To investigate the effect of OICR-9429 on apoptosis in BCa cells, we performed flow cytometry and caspase 3/7 assays. The data showed no obvious apoptotic cells after treatment with OICR-9429 for 24 h, but the apoptotic rate was significantly increased at 72 h (Fig. 3a, SFig. 3a, b and c). Similarly, caspase 3/7 activity was also upregulated in the cells treated with OICR-9429 at 72 h (SFig. 3d). These data suggest that OICR-9429 enhances apoptosis of BCa cells in a time-dependent and dose-dependent manner.

Cisplatin-based chemotherapy is the first-line therapeutic treatment in BCa, but it has limited therapeutic response and serious side effects, especially nephrotoxicity [5]. We then evaluated whether combining OICR-9429 could enhance antitumour activity and reduce the dose of cisplatin. First, three BCa cells were treated with various concentrations of cisplatin combined with three concentrations of OICR-9429 (0, IC_50_, 2 IC_50_). Compared with treatment with cisplatin alone, combined OICR-9429 therapy obviously enhanced the cytotoxicity of cisplatin in a dose-dependent manner (Fig. 3b). In addition, according to the Chou-Talalay method [27], we calculated the combination index (CI) values to evaluate the combined effect of OICR-9429 and cisplatin on BCa cells. We discovered that OICR-9429 promoted cisplatin cytotoxicity through synergistic effect in T24 and UM-UC-3 cells (CI < 1, SFig. 4a). Given that cisplatin is a DNA-damaging drug, we next performed comet assay to detect the DNA damage level in single BCa cells. Interestingly, we found that OICR-9429 obviously increased the DNA damage level caused by cisplatin treatment in BCa cells (Fig. 3c and d). Next, we examined apoptosis by staining cells with Annexin V and propidium iodide (PI). Three BCa cells were treated with 2 concentrations of OICR-9429 (IC_50_, 2 IC_50_), 2 concentrations of cisplatin (1, 2 μg/mL) and a combination of IC_50_ OICR-9429 and low-dose cisplatin (1 μg/mL). Flow cytometry indicated that three BCa cells treated with a combination of low-dose OICR-9429 and cisplatin exhibited the highest apoptotic rates after 60 h of treatment (Fig. 3e and f, SFig. 4b). However, OICR-9429 showed no obvious synergistic cytotoxic effect with gemcitabine (SFig. 4c). Collectively, we found that OICR-9429 increased cell apoptosis and promoted cisplatin chemosensitivity in BCa cells.

### OICR-9429 suppresses tumour growth and enhances cisplatin efficacy in bladder cancer cells in vivo

**Fig. 4:**
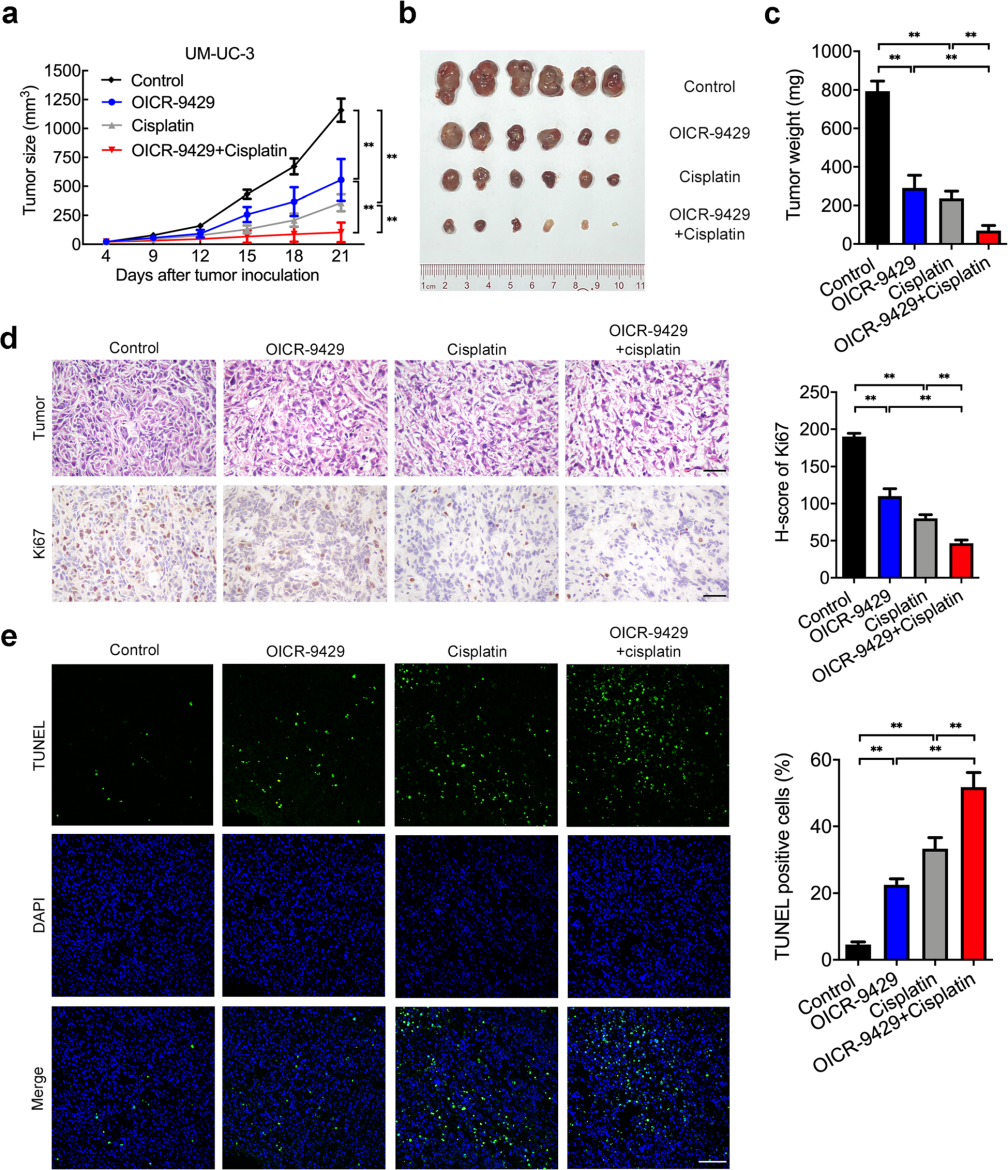
OICR-9429 suppresses tumour growth and enhances cisplatin efficacy in bladder cancer cells in vivo*.* **a.** The volume of tumours in the indicated groups was measured every 3 days. The average tumour volume is shown as the mean ± SD of six mice. **b.** Representative images of dissected tumours treated with control solvent, OICR-9429 (60 mg/kg), cisplatin (4 mg/kg), or a combination of small dose OICR-9429 (30 mg/kg) and cisplatin (2.5 mg/kg). **c.** Tumour weights were measured after the tumours were surgically dissected. **d.** The expression of Ki67 in the tumour was examined by IHC. Histogram shows the H-score of Ki67 IHC in each group. Scale bars, 50 μm (black). **e.** Tumour apoptosis was detected by TUNEL assay. Histogram shows the proportion of TUNEL-positive cells in each group. Scale bars, 100 μm (white). *p < 0.05; **p < 0.01

We next determined the effect of OICR-9429 on tumorigenesis and cisplatin therapy response in vivo. As shown in Fig. 4a, b and c, single OICR-9429 (60 mg/kg) or cisplatin (4 mg/kg) treatment suppressed tumour growth in vivo and produced a smaller volume of tumours than the control group. Interestingly, combination therapy of a small dose of OICR-9429 (30 mg/kg) and cisplatin (2.5 mg/kg) produced significantly slower tumour growth and the smallest tumours compared to those treated with either agent alone, suggesting that OICR-9429 enhanced tumour sensitivity to cisplatin. To assess the toxicity of these drugs to organs, we performed H&E staining of the kidney, heart, lung and liver in mice in these four groups. We found that high-dose cisplatin (4 mg/kg) treatment caused renal tubular injury, which was consistent with the nephrotoxicity of cisplatin, but no obvious renal tubular injury was observed in cells treated with either OICR-9429 alone or a combination of OICR-9429 and cisplatin (SFig. 5). No significant morphological changes in the heart, lung or liver were observed in the treatment groups. Consistent with the in vivo results, immunohistochemistry staining of the proliferation marker Ki67 and TUNEL assays revealed that tumours derived from the combined treatment group had the lowest proliferation activity and the highest apoptotic rate compared with the single treatment of OICR-9429 or cisplatin or the control (Fig. 4d and e). Collectively, these data strongly indicated that targeting WDR5 not only suppressed tumour proliferation and enhance the efficacy of cisplatin for BCa cells in vivo but also reduced the toxicity and side effects for normal tissues.

### OICR-9429 suppresses the metastatic behaviour of bladder cancer cells

**Fig. 5:**
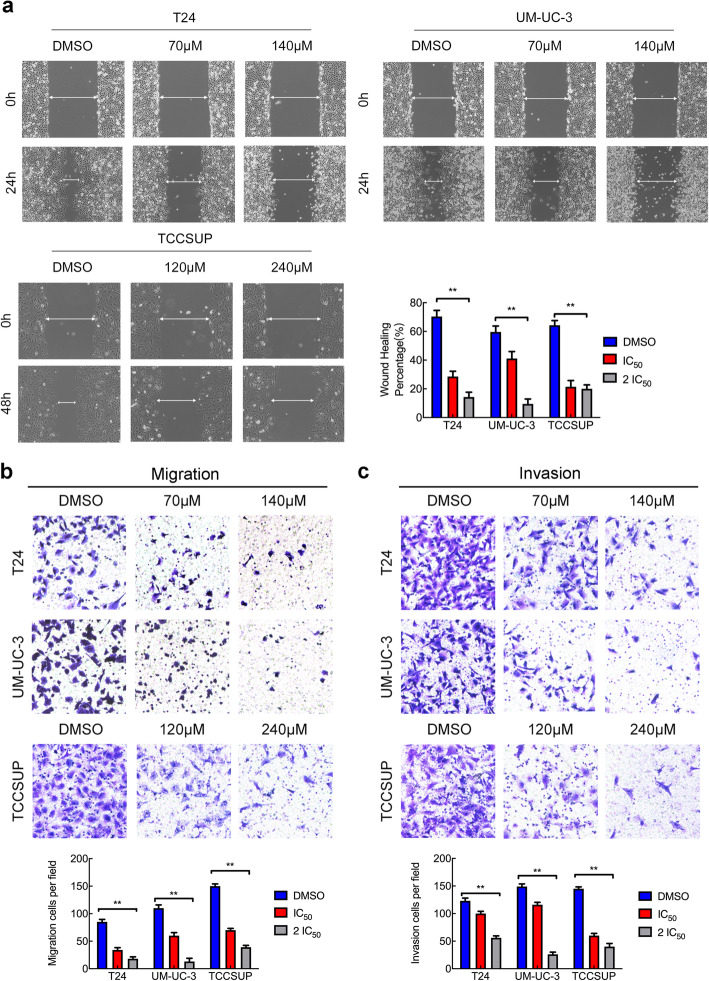
OICR-9429 suppresses the metastatic behaviour of bladder cancer cells. **a.** Representative images and histogram analysis of wound-healing assays using three BCa cells showed cell motility after treatment with two OICR-9429 doses or DMSO. **b. c.** Representative images of migration (**b**) and invasion (**c**) assays using three BCa cell lines showing cell migration and invasion after treatment with two OICR-9429 doses or DMSO. A histogram analysis of migrated or invaded cell counts is shown. *p < 0.05 and **p < 0.01

To determine the effect of OICR-9429 on cell motility and tumour metastasis, we treated three BCa cells with OICR-9429 for 24 h and then conducted wound healing assays, cell migration and cell invasion. Wound healing assays showed that inhibition of WDR5 reduced the migratory speed of the three BCa cell lines (Fig. 5a). Moreover, we found that OICR-9429 treatment significantly decreased the migration and invasion of the three BCa cell lines (Fig. 5b and c). These data suggested that OICR-9429 suppressed the motility and metastatic behaviour of BCa cells independent of apoptosis and proliferation.

### The target genes for OICR-9429 are identified in bladder cancer

**Fig. 6:**
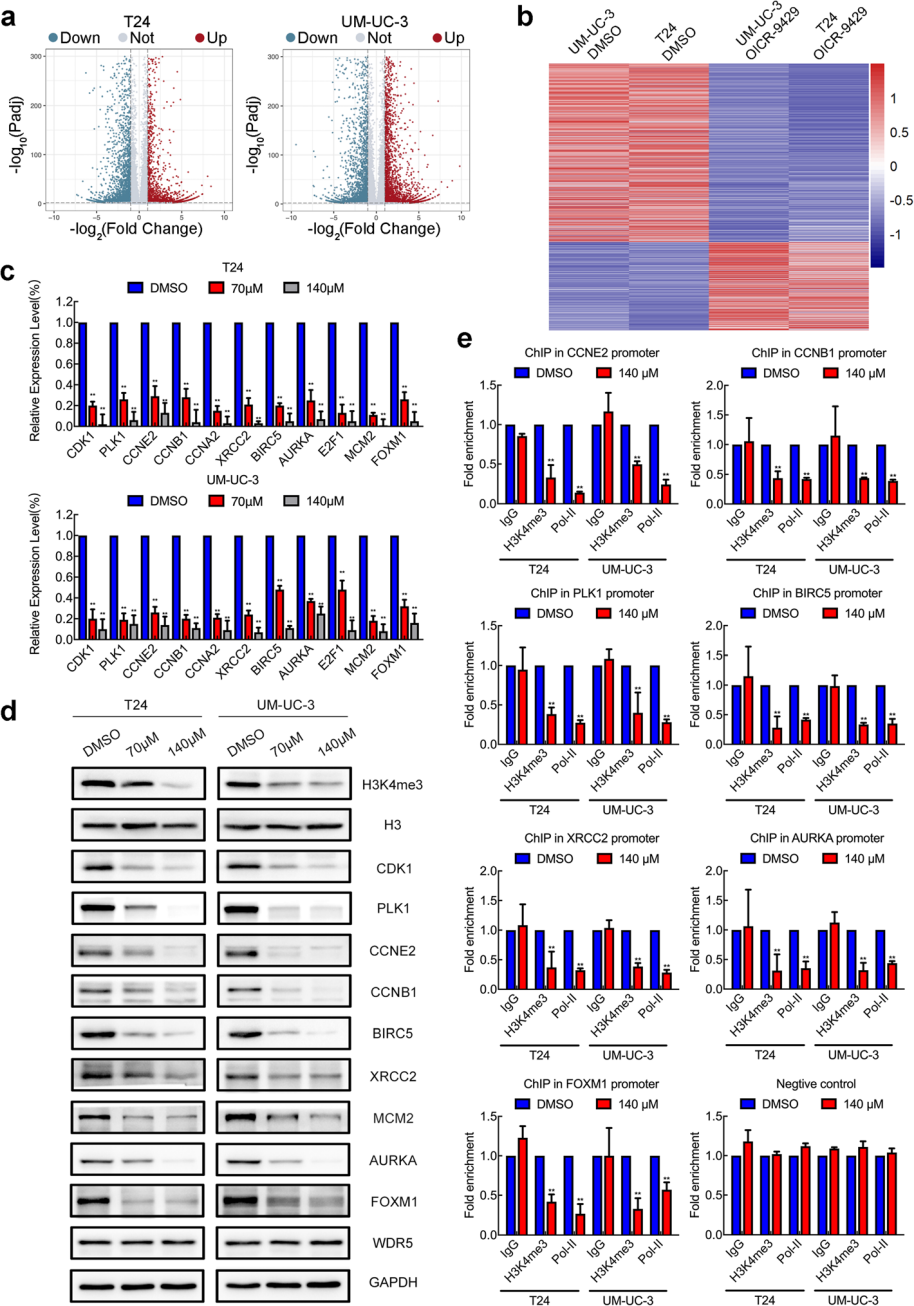
Target genes of OICR-9429 are identified in bladder cancer. **a.** Volcano plot showing the differentially expressed genes in RNA sequencing in T24 and UM-UC-3 cells treated with DMSO or 140 μM OICR-9429 for 48 h. **b.** A heatmap representing both downregulated mRNA expression levels in T24 and UM-UC-3 cells treated with OICR-9429. **c.** The differentially expressed genes in the RNA sequencing were verified in T24 and UM-UC-3 cells by qRT-PCR. **d.** The expression of OICR-9429 target genes in T24 and UM-UC-3 cells was detected by Western blots. GAPDH and histone H3 were used as internal controls. **e.** ChIP-qPCR analysis of H3K4 tri-methylation and Pol-II status in the promoters of target genes after treatment with DMSO or 140 μM OICR-9429 for 48 h in T24 and UM-UC-3 cells. Values were normalized to the DMSO groups. *p < 0.05 and **p < 0.01

To explore the molecular mechanism underlying OICR-9429-induced WDR5 inhibition in BCa cells, genome-wide RNA sequencing was conducted to compare gene expression profiles between OICR-9429-treated T24 and UM-UC-3 cells and their control cells. Interestingly, 3747 and 4527 genes, respectively, were differentially expressed in T24 and UM-UC-3 cells after treatment with OICR-9429 (Fig. 6a). Given that MLL1-WDR5 is a transcriptionally activated complex, we focused on 956 genes that were downregulated in both T24 and UM-UC-3 cells (Fig. 6b, SFig. 6a). Furthermore, Gene Ontology (GO) analysis revealed that OICR-9429 participated in serial biological processes, such as cell cycle, DNA repair, apoptosis and migration (SFig. 6b). Then, we validated the expression of some key genes in T24, UM-UC-3, and TCCSUP cells treated with OICR-9429 by qRT-PCR. As expected, some genes related to the cell cycle, such as CDK1, PLK1, CCNE2, CCNB1, CCNA2, AURKA, and E2F1, genes related to apoptosis and DNA repair, such as BIRC5, XRCC2, AURKA, E2F1, and MCM2, and genes related to metastasis, such as AURKA and FOXM1, were significantly downregulated in OICR-9429-treated cells (Fig. 6c, SFig. 6c). Furthermore, the protein expressions of CDK1, PLK1, CCNE2, CCNB1, BIRC5, XRCC2, MCM2, AURKA, and FOXM1 were consistent with the mRNA level from Western blots (Fig. 6d, SFig. 6d and e). Furthermore, TCGA analysis revealed that WDR5 was markedly, positively correlated with these genes in BCa (SFig. 7). To further explore whether OICR-9429 directly affected the target genes, we conducted ChIP assays in T24 and UM-UC-3 cells treated with OICR-9429 for 48 h. Interestingly, we found that OICR-9429 treatment decreased H3K4me3 and RNA polymerase-II levels at the promoter regions of CCNE2, CCNB1, PLK1, BIRC5, XRCC2, AURKA, and FOXM1 in T24 and UM-UC-3 cells but not in the negative control group or other genes (Fig. 6e, SFig. 8). Our data revealed that downregulation of these genes was directly regulated by OICR-9429-mediated blockade of WDR5. Taken together, we found that OICR-9429 targeted the WDR5-MLL1 complex to downregulate the cell cycle-, apoptosis-, DNA repair- and metastasis-related genes by mediating H3K4me3 of gene promoters.

### WDR5 positively correlates with PD-L1, and OICR-9429 suppresses immune evasion by blocking PD-L1 activation

**Fig. 7:**
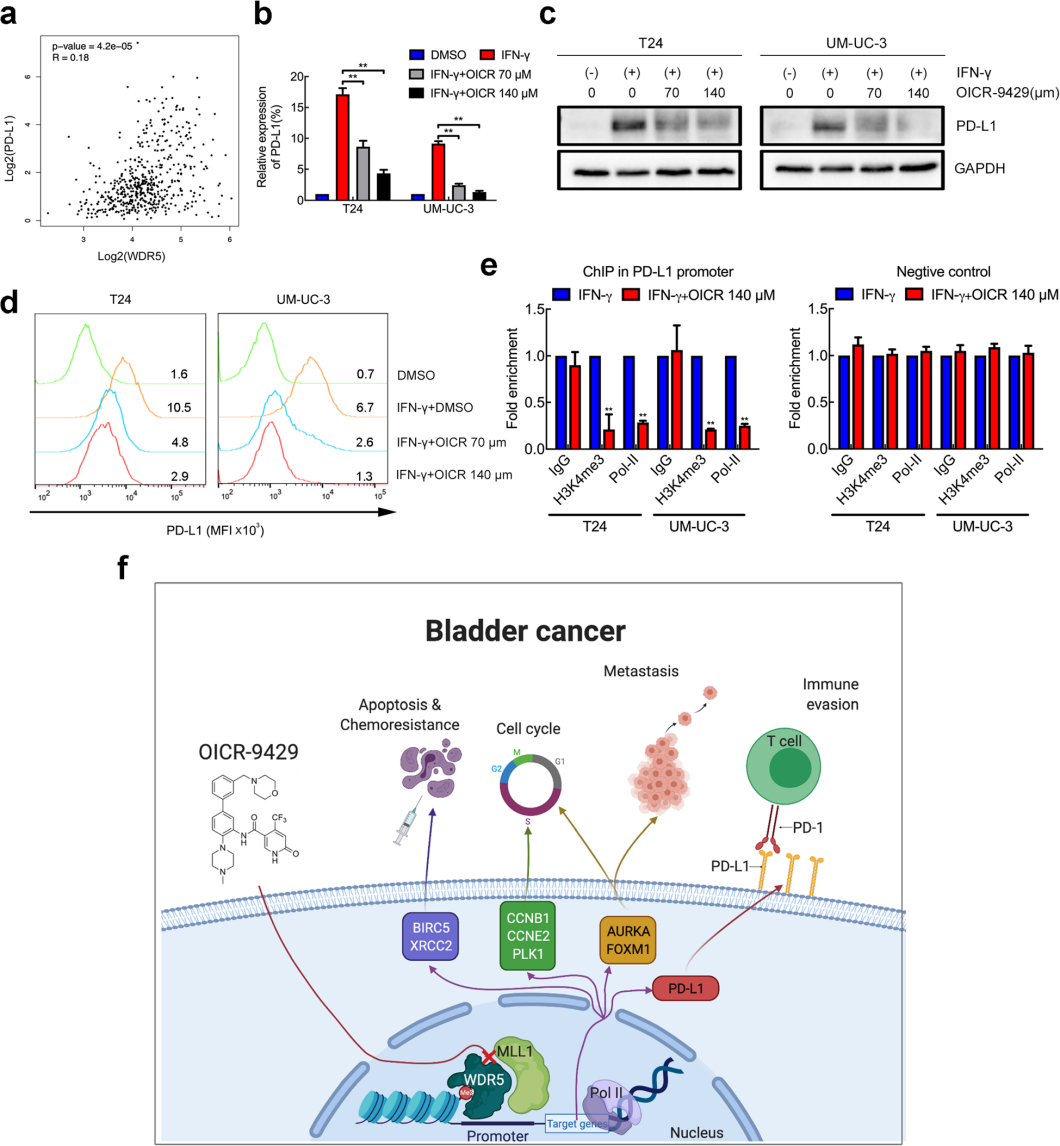
WDR5 positively correlates with PD-L1, and OICR-9429 suppresses immune evasion by blocking PD-L1 activation. **a.** Pearson correlation between the expression of WDR5 and PD-L1 in the TCGA cohort. **b. c.** Relative mRNA (**b**) and protein (**c**) expression of PD-L1 in two BCa cell lines treated with DMSO, IFN-γ, IFN-γ + IC_50_ OICR-9429, and IFN-γ + 2 IC_50_ OICR-9429. GAPDH was used as the internal control. **d.** Flow cytometry verified that OICR-9429 reduced PD-L1 expression induced by IFN-γ treatment in two BCa cell lines. **e.** ChIP-qPCR analysis of H3K4 tri-methylation and Pol-II status in the PD-L1 promoter after treatment with IFN-γ or IFN-γ + 2 IC_50_ OICR-9429 for 48 h in T24 and UM-UC-3 cells. Values were normalized to the IFN-γ group. **f.** A schematic model of the mechanism underlying the role of OICR-9429 in BCa proliferation, chemosensitivity, metastasis and immune evasion. *p < 0.05 and **p < 0.01

Inhibition of epigenetic modifications such as HDAC, EZH2, and PARP has been recently proven to be associated with immune evasion and immunomodulatory effects in BCa and other malignant tumours [28–30]. To explore whether targeting the MLL1-WDR5 complex could regulate PD-L1 expression, we analysed TCGA data. Interestingly, WDR5 expression was notably positively correlated with PD-L1 (Fig. 7a). Wild-type T24 and UM-UC-3 cells expressed PD-L1 at a relatively low level without stimulation. Hence, we treated T24 and UM-UC-3 cells with IFN-γ (100 ng/mL, 48 h) to simulate the tumour microenvironment and increase PD-L1 expression. Strikingly, we found that OICR-9429 significantly reduced the expression of PD-L1 induced by IFN-γ in a dose-dependent manner at both the RNA and protein levels (Fig. 7b and c). Moreover, IFN-γ treatment increased the rate of PD-L1-positive T24 and UM-UC-3 cells, and flow cytometry showed that OICR-9429 treatment blocked this PD-L1 upregulation (Fig. 7d). To investigate the mechanism of OICR-9429 on PD-L1 regulation, ChIP assays in T24 and UM-UC-3 cells were performed. As a result, inhibition of the MLL1-WDR5 complex using OICR-9429 decreased the H3K4me3 and RNA polymerase-II levels in the PD-L1 promoter but not in the negative controls (Fig. 7e). Collectively, these data indicated that WDR5 had positive correlation with PD-L1 and that OICR-9429 suppressed immune evasion by blocking PD-L1 activation in BCa cells.

## Discussion

Muscle-invasive BCa is a lethal disease with high metastasis, low chemosensitivity, and poor prognosis [3, 5]. Hence, it is urgent to develop novel approaches to block uncontrolled proliferation and metastasis and enhance chemosensitivity for BCa. A recent study identified some key oncogenes and tumour suppressors in the regulation of proliferation, metastasis and chemoresistance of BCa, such as PTBP1, hnRNPK, NONO, REDD1, lncRNA LBCS, and DANCR [21, 22, 31–34]. However, specific inhibitors of these targets have not been developed, and there is still a long way to go from concept to reality. Several studies have proven that some small molecule drugs that are used in other malignancies, such as PI3K inhibitors, CDK4/6 inhibitors, FGFR inhibitors and AR inhibitors, show promising inhibitory effects on BCa [35–38]. Therefore, gene-based targeted therapy will become the main treatment for advanced BCa, and it is necessary to investigate and develop more molecular drugs that target vital signalling in BCa. Here, we first present data supporting the basis for a novel approach to treat advanced BCa. OICR-9429, a WDR5 inhibitor, holds promise as a therapy for BCa.

Histone modification is proven to be one of the most important epigenetic regulation mechanisms and druggable targets [16]. The methylation of histone H3K4 has a major impact on regulation of genes expression that are critical for normal biology and diseases, including cancer [11]. Through protein complexes formation with H3K4 methyltransferases, such as SET1A/B and MLL1–4, WDR5 plays an essential role in H3K4me3 and transcriptional activation of target genes involved in pluripotency, development, and cancer, such as MLL-rearranged leukaemia [39]. Furthermore, WDR5 has been well established as an oncogene and is closely associated with the tumorigenesis and progression of multiple cancers, such as leukaemia and bladder, prostate, and colon cancer [12–15]. Recent studies indicate that WDR5 also interacts with long, noncoding RNAs (lncRNAs), such as HOXD-AS1 and HOTTIP, to facilitate gene transcription in cancer via lncRNA-guided histone H3K4 trimethylation [23, 40, 41]. Therefore, WDR5 may act as a promising target in BCa treatment. The specific structural feature of the MLL/WDR5 complex is a novel drug target. OICR-9429, a high affinity small molecule compound, competitively blocks WDR5 interaction with MLL protein via binding the central peptide-binding pocket of WDR5 [17]. OICR-9429 has shown anticancer efficacy by inhibiting cell vitality in non-MLL-rearranged leukaemia and colon and pancreatic cancer [14, 18]. However, the biological function affected by OICR-9429 in BCa remains unknown. In this study, we first discovered that OICR-9429 inhibited the proliferation of BCa cells by blocking the G1/S phase transition. Furthermore, OICR-9429 inhibited cell motility and metastatic behaviour but enhanced cell apoptosis and cisplatin chemosensitivity in BCa cells. Additionally, OICR-9429 suppressed tumour growth and promoted the chemosensitivity of cisplatin in BCa cells in vivo, suggesting that combined therapy with OICR-9429 and cisplatin could improve the curative effect and reduce side effects. Therefore, targeting WDR5 with OICR-9429 is multipotent and promotes anticancer therapy for BCa.

Although OICR-9429 is a WDR5 inhibitor, the genes affected by OICR-9429 in BCa remain largely unknown. Here, we first performed RNA sequencing to investigate the landscape of genes regulated by OICR-9429-mediated blockade of WDR5. Interestingly, 956 genes were downregulated in both T24 and UM-UC-3 cells. Through qPCR, Western blot and ChIP verification, we found that CCNB1, CCNE2, PLK1, BIRC5, XRCC2, AURKA, and FOXM1 were the direct targets of OICR-9429. BIRC5 and XRCC2 regulate antiapoptosis and DNA repair, contributing to cancer chemoresistance [42, 43]. CCNB1, CCNE2, AURKA, and FOXM1 regulate cell cycle transition, resulting in uncontrolled proliferation [44, 45]. Emerging evidence shows that AURKA and FOXM1 are multifunctional genes that promote proliferation, chemoresistance and metastasis via different mechanisms [46–48]. This finding was not only consistent with our previous study of WDR5 in BCa [13, 49], but also expanded insight into the mechanism by which OICR-9429 targets WDR5.

Immune checkpoint inhibitors (ICIs) against PD-1 and its ligand, PD-L1, which is an emerging treatment for BCa immunotherapy, have received the most attention [6]. ICIs offer an alternative solution for MIBC patients who were previously ineligible for cisplatin-based chemotherapy or intolerable due to toxicity. Due to the heterogeneity of tumour cells and the complexity of the tumour immune microenvironment, most patients with advanced BCa have no response solely to immunotherapy (objective response rates from 17.8 to 24%) [6]. Therefore, combined treatments with ICIs and targeted therapies are urgently required to improve current immunotherapy strategies [50]. Previous studies showed that epigenetic modifications, such as HDAC6, EZH2, PRC2, and H3K27me3, played a vital role in immune microenvironment regulation [51–53]. In this study, we demonstrated that WDR5 markedly correlated with PD-L1 expression in BCa and that OICR-9429 suppressed immune evasion by blocking PD-L1 activation induced by IFN-γ. Moreover, ChIP assays further confirmed that OICR-9429 directly reduced the enrichment of histone H3K4 tri-methylation and RNA polymerase-II in the PD-L1 promoter region, leading to the inhibition of PD-L1. Taken together, these data indicated that OICR-9429 may be a promising drug for combining immunotherapy to improve clinical outcomes since it inhibits PD-L1 expression in BCa cells.

## Conclusions

In summary, our novel discovery showed that the WDR5 inhibitor, OICR-9429, suppressed proliferation, metastasis and PD-L1-based immune evasion while enhancing apoptosis and cisplatin chemosensitivity in BCa by blocking the WDR5-MLL complex mediating H3K4me3 of target genes (Fig. 7f). Therefore, our findings provide sufficient evidence that OICR-9429 might be a multipotent anticancer therapy, and could offer novel therapeutic schemes for bladder cancer patients together with cisplatin and/or immune-checkpoint inhibitors.

## Supplementary Information

**Additional file 1: Supplementary Fig. 1.** The expression of WDR5 in age, gender, T-stage, N-stage and Non-muscle invasive bladder cancer (NMIBC) history features in TCGA cohort. **Supplementary Fig. 2.** The images of EdU assay of three BCa cells treated with two doses of OICR-9429 or DMSO. **Supplementary Fig. 3.** OICR-9429 increases apoptosis of bladder cancer cells. **Supplementary Fig. 4.** OICR-9429 increases cisplatin chemosensitivity of bladder cancer cells but not gemcitabine. **Supplementary Fig. 5.** H&E staining of kidney, liver, lung, and heart from the mice in indicated groups. **Supplementary Fig. 6.** The target genes of OICR-9429 are identified in bladder cancer. **Supplementary Fig. 7.** Pearson correlations between the expression of WDR5 and CDK1, PLK1, CCNE2, CCNB1, BIRC5, XRCC2, MCM2, AURKA and FOXM1 in TCGA cohort. **Supplementary Fig. 8.** OICR-9429 treatment did not affect H3K4me3 and RNA polymerase-II levels on the promoter regions of CDK1 and MCM2 in UM-UC-3 and T24 cells. **Supplementary Fig. 9.** Original images of western blotting. **Supplementary Table 1.** Univariate and multivariate analysis of factors associated with overall survival in 345 cases of bladder cancer from TCGA cohort. **Supplementary Table 2.** Primers used in qPCR. **Supplementary Table 3.** Primers used in ChIP-qPCR.

## Data Availability

The datasets used and/or analysed during the current study are available from the corresponding author on reasonable request.

## Abbreviations

BCa: Bladder cancer
WDR5: WD repeat-containing protein 5
H3K4me3: Histone H3 lysine 4 tri-methylation
qPCR: Quantitative polymerase chain reaction
STR: Short tandem repeat
IC_50_: Half maximal inhibitory concentration
CIs: Confidence intervals
CI: Combination index
PD-1: Programmed death-1
PD-L1: Programmed death-ligand 1
ChIP: Chromatin immunoprecipitation
IFN-γ: Interferon-γ
MLL1: Mixed-lineage leukemia 1
NMIBC: Non-muscle invasive bladder cancer
MIBC: Muscle invasive bladder cancer
FDA: US food and drug administration
TCGA: The cancer genome atlas
GEO: Gene expression omnibus
OS: Overall survival
GO: Gene ontology
LncRNA: Long noncoding RNA
ICI: Immune checkpoint inhibitors
