# Supplementary material for: Targeting WD repeat domain 5 enhances chemosensitivity and inhibits proliferation and programmed death-ligand 1 expression in bladder cancer
Source: J Exp Clin Cancer Res. 2021 Jun 21;40:203. doi: 10.1186/s13046-021-01989-5 (PMC8215817; doi:10.1186/s13046-021-01989-5)
Supplement: Supplementary file 1 — Additional file 1: Supplementary Fig. 1. The expression of WDR5 in age, gender, T-stage, N-stage and Non-muscle invasive bladder cancer (NMIBC) history features in TCGA cohort. Supplementary Fig. 2. The images of EdU assay of three BCa cells treated with two doses of OICR-9429 or DMSO. Supplementary Fig. 3. OICR-9429 increases apoptosis of bladder cancer cells. Supplementary Fig. 4. OICR-9429 increases cisplatin chemosensitivity of bladder cancer cells but not gemcitabine. Supplementary Fig. 5. H&E staining of kidney, liver, lung, and heart from the mice in indicated groups. Supplementary Fig. 6. The target genes of OICR-9429 are identified in bladder cancer. Supplementary Fig. 7. Pearson correlations between the expression of WDR5 and CDK1, PLK1, CCNE2, CCNB1, BIRC5, XRCC2, MCM2, AURKA and FOXM1 in TCGA cohort. Supplementary Fig. 8. OICR-9429 treatment did not affect H3K4me3 and RNA polymerase-II levels on the promoter regions of CDK1 and MCM2 in UM-UC-3 and T24 cells. Supplementary Fig. 9. Original images of western blotting. Supplementary Table 1. Univariate and multivariate analysis of factors associated with overall survival in 345 cases of bladder cancer from TCGA cohort. Supplementary Table 2. Primers used in qPCR. Supplementary Table 3. Primers used in ChIP-qPCR. [file 13046_2021_1989_MOESM1_ESM.docx]

**Targeting WD repeat domain 5 enhances chemosensitivity and inhibits proliferation and programmed death-ligand 1 expression in bladder cancer**

Jingtong Zhang^1,2,3 #^, Qianghua Zhou^1,2,3 #^, Keji Xie^4, #^, Liang Cheng^1,2,3^, Shengmeng Peng^1,2,3^, Ruihui Xie^1,2,3^, Lixuan Liu^5^, Yangjie Zhang^1,2,3^, Wen Dong^1,3^, Jinli Han^1,3^, Ming Huang^1,2,3^, Yuelong Chen^1,6^, Tianxin Lin^1,2,3,7, *^, Jian Huang^1^^,2,3 *^, Xu Chen^1,2,3 *^.

1 Department of Urology, Sun Yat-sen Memorial Hospital, Sun Yat-sen University, Guangzhou, China

2 Guangdong Provincial Key Laboratory of Malignant Tumor Epigenetics and Gene Regulation, Sun Yat-Sen Memorial Hospital, Sun Yat-Sen University, Guangzhou, China

3 Guangdong Provincial Clinical Research Center for Urological Diseases

4 Department of Urology, Guangzhou First People's Hospital, Guangzhou, China

5 Department of Endocrinology, Sun Yat-sen Memorial Hospital, Sun Yat-sen University, Guangzhou, China

6 Department of Urology, The 1st Affiliated Hospital of Kunming Medical University, Kunming, China

7 Department of Urology, The Affiliated Kashi Hospital, Sun Yat-sen University, Kashi, China

^#^ Jingtong Zhang, Qianghua Zhou and Keji Xie contributed equally to this work.

***Correspondence**

Xu Chen, Tianxin Lin and Jian Huang, Department of Urology, Sun Yat-sen Memorial Hospital, 107th Yanjiangxi Road, Guangzhou, China. E-mail: chenx457@mail.sysu.edu.cn, lintx@mail.sysu.edu.cn, huangj8@mail.sysu.edu.cn


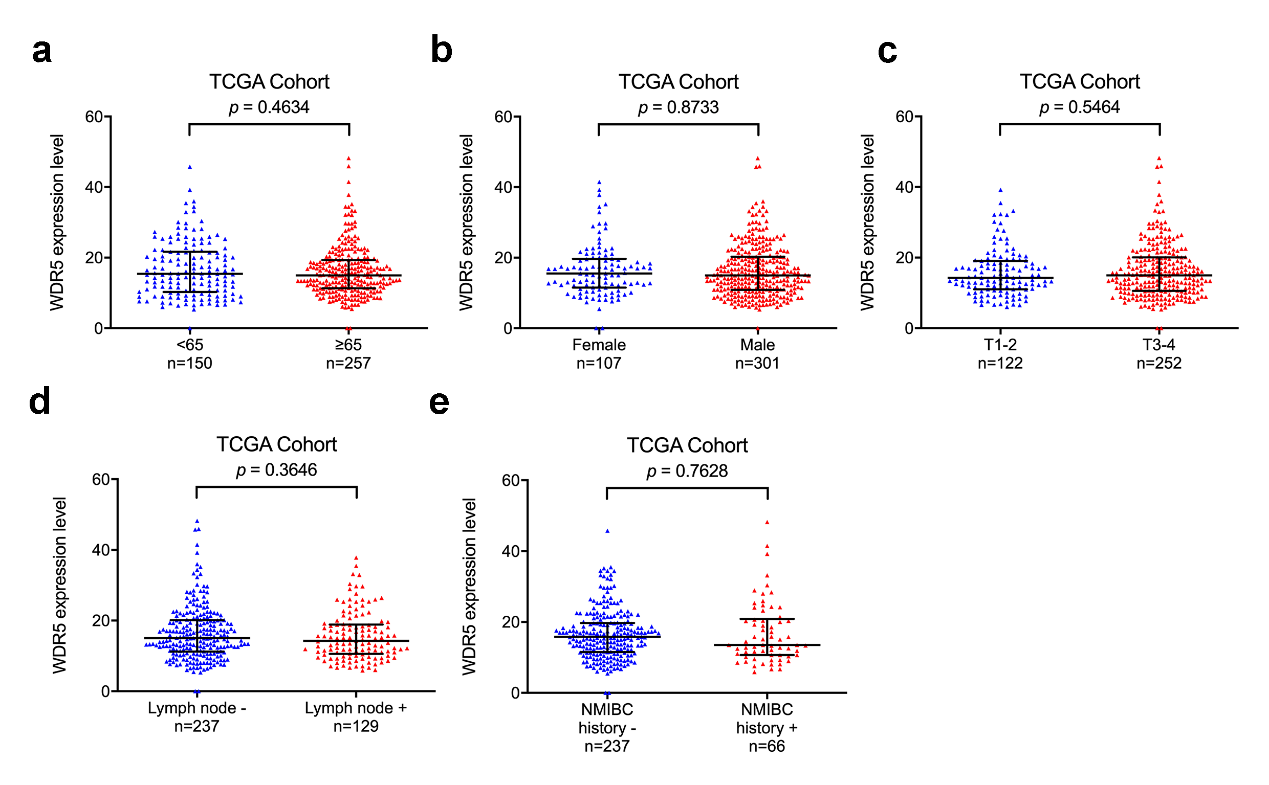


**Supplementary Figure 1. The expression of WDR5 in age (a), gender (b), T-stage (c), N-stage (d) and Non-muscle invasive bladder cancer (NMIBC) history features (e) in TCGA cohort.**

**
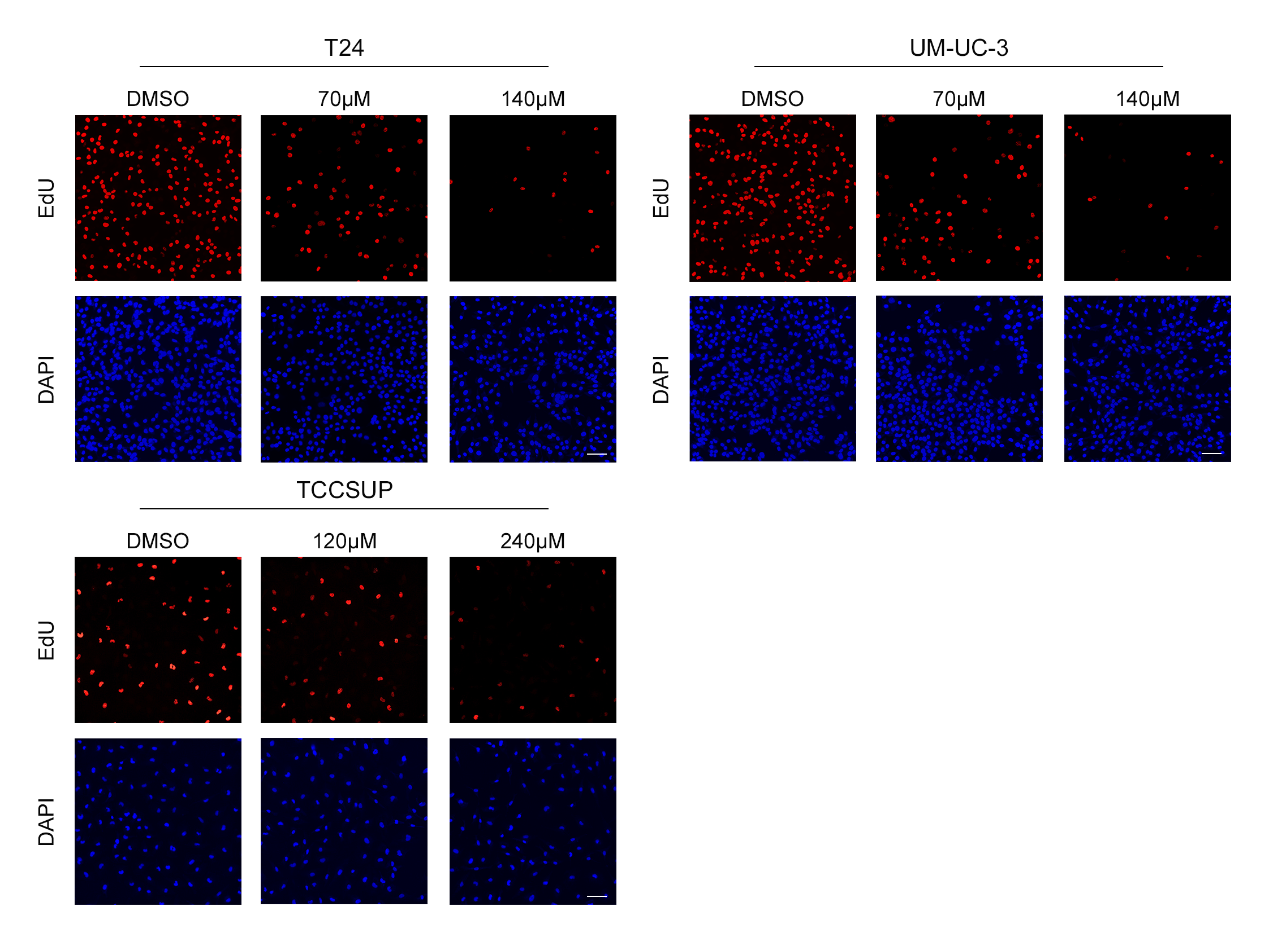
**

**Supplementary Figure 2. The images of EdU assay of three BCa cells treated with two doses of OICR-9429 or DMSO.** Blue indicated nucleus; red indicated S-phase cells. Scale bars, 50 µm (white).


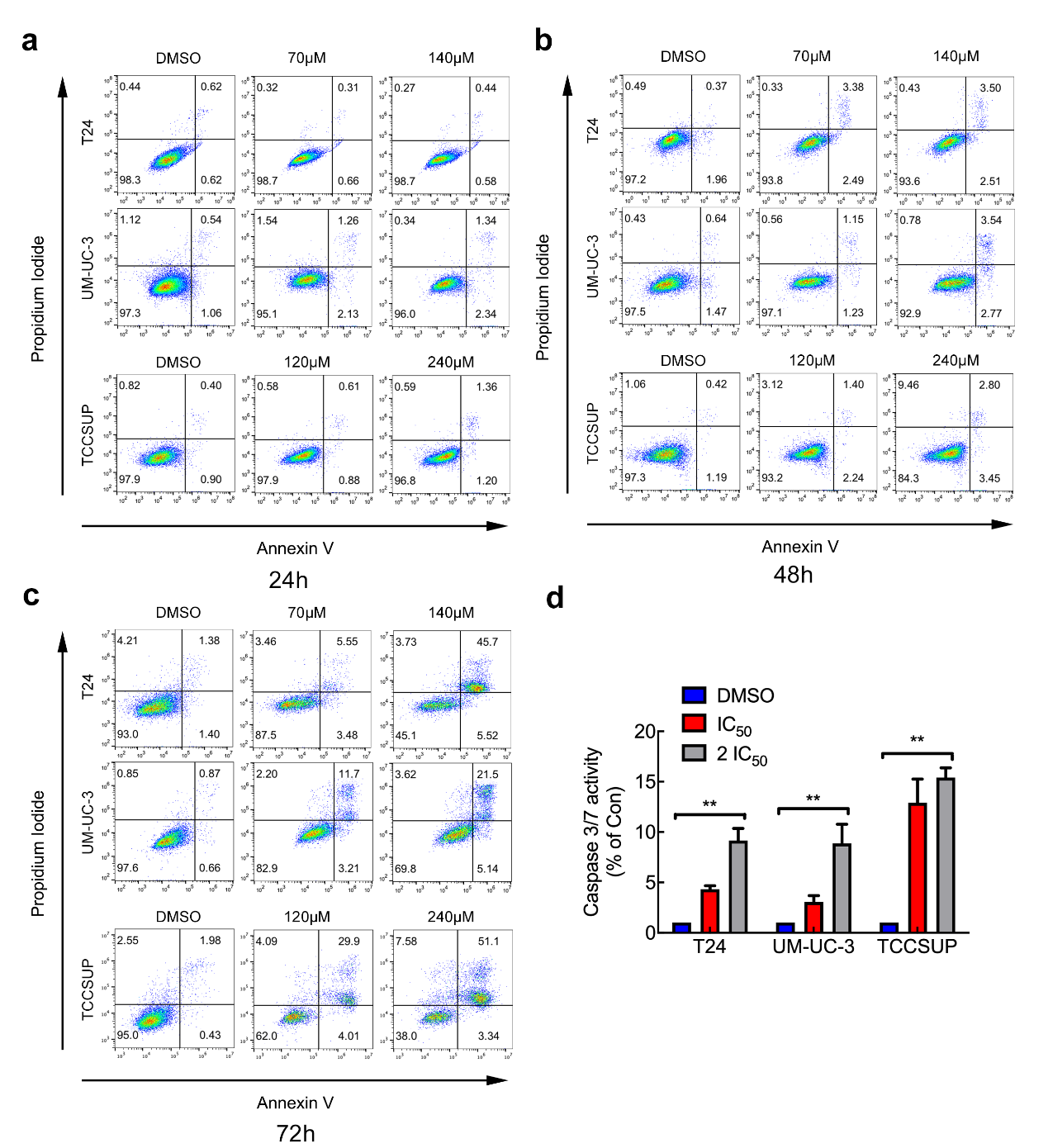


**Supplementary Figure 3. OICR-9429 increases apoptosis of bladder cancer cells.**

**a-c.** The apoptosis analysis of three BCa cells treated with two doses of OICR-9429 or DMSO for 24 h (**a**), 48 h (**b**), 72 h (**c**). **d.** The histogram analysis showed the caspase 3/7 activity was upregulated in three BCa cells treated with OICR-9429 for 72 h. *p < 0.05 and **p < 0.01.

**
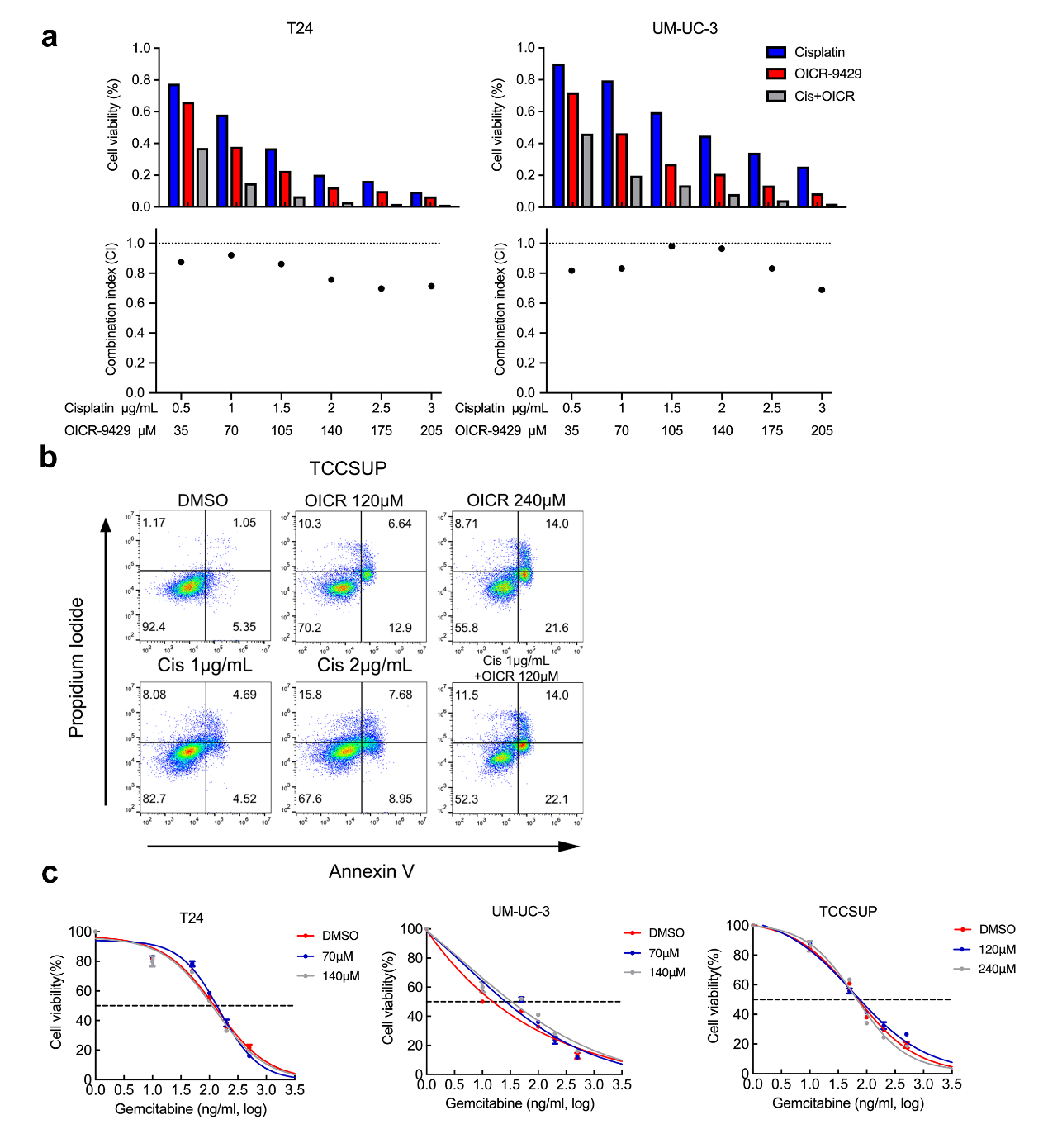
Supplementary Figure 4. OICR-9429 increases cisplatin chemosensitivity of bladder cancer cells but not gemcitabine.**

**a.** Cell viability (Top) and combination index values (Bottom) of OICR-9429 and cisplatin individual or combined treatments in T24 and UM-UC-3 cells. **b.** The apoptosis analysis of TCCSUP cells treated with 2 concentrations of OICR-9429 (IC_50_, 2IC_50_), 2 concentrations of cisplatin (1, 2 μg/mL) and a combining treatment with IC_50_ OICR-9429 and low-dose cisplatin (1 μg/mL) for 72 h. **c.** The cell viability of OICR-9429 combined with gemcitabine in three BCa cells by MTT assay.


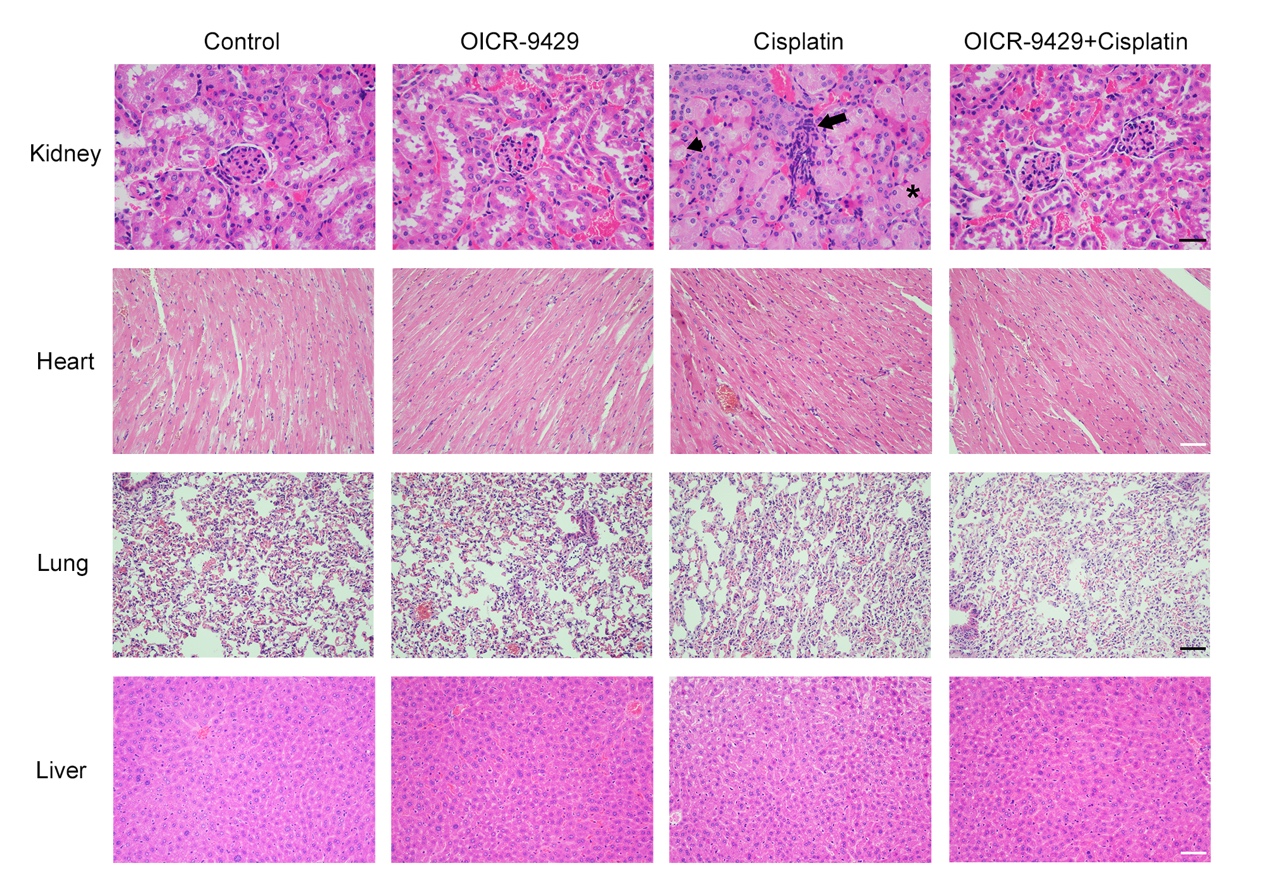


**Supplementary Figure 5. H&E staining of kidney, liver, lung, and heart from the mice in indicated groups.**

The tumor-bearing mice were received control solvent, OICR-9429 (60 mg/kg), cisplatin (4 mg/kg), or a combination of small dose OICR-9429 (30 mg/kg) and cisplatin (2.5 mg/kg), respectively. Asterisk indicated renal tubular casts; arrow indicated infiltrated inflammatory; arrow head indicated vacuolar degeneration and necrosis. Scale bars, 50 µm (kidney, black), 100 µm (other organs, white/black).

**
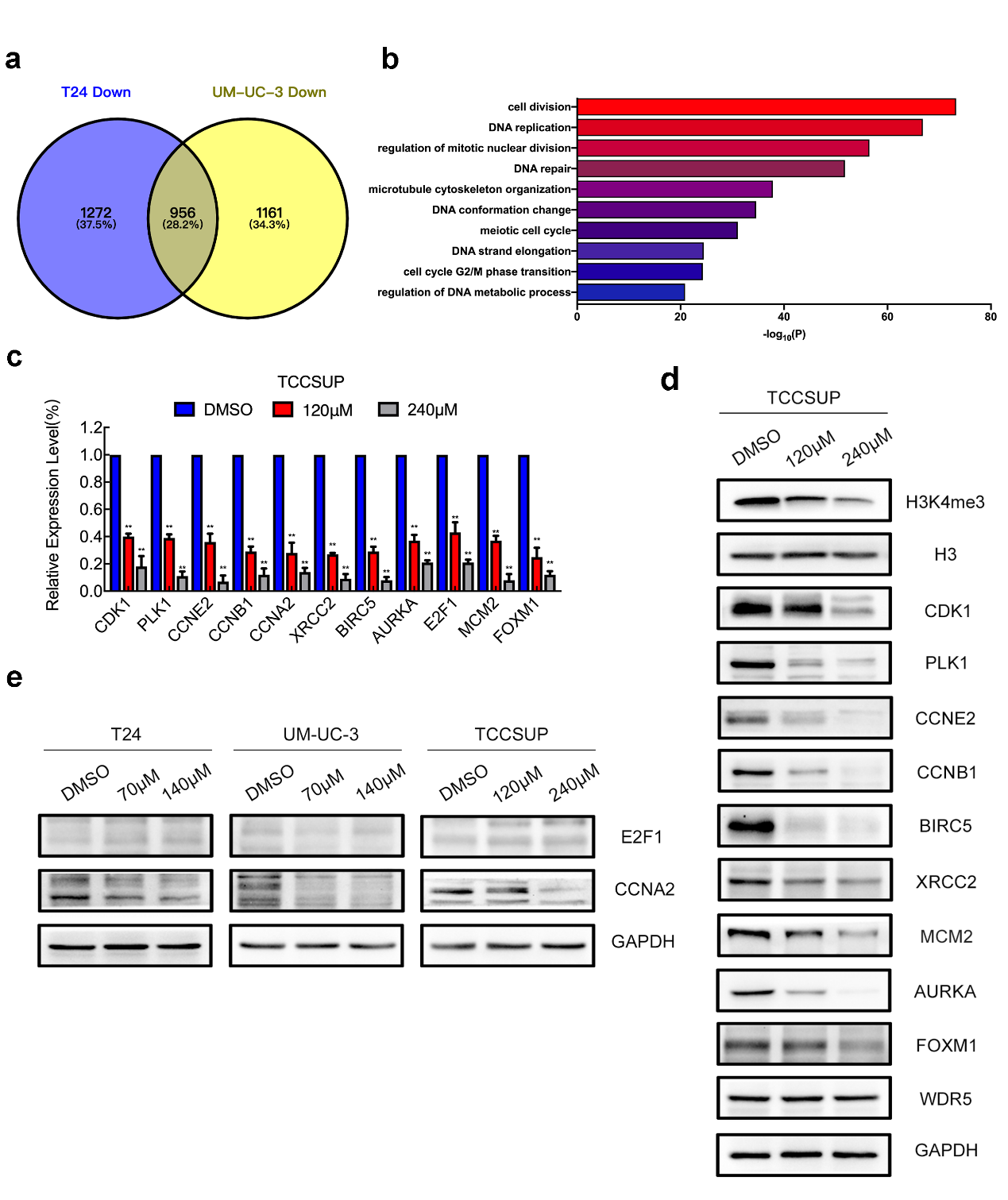
**

**Supplementary Figure 6. The target genes of OICR-9429 are identified in bladder cancer.**

**a.** Venn image showed that 956 genes were down-regulated in both T24 and UM-UC-3 cells. **b.** Gene ontology (GO) analysis revealed that OICR-9429 participates in serial genes related to cell cycle, DNA repair, apoptosis and migration. **c.** The differentially expressed genes in the RNA sequencing were verified in TCCSUP cells by qRT-PCR. **d.** The expression of OICR-9429 target genes were detected by western blotting in TCCSUP cells. GAPDH and Histone H3 were used as the internal control. **e.** E2F1 protein was not detected by western blotting in three BCa cells, and CCNA2 protein expression was not exactly consistent with mRNA level alteration. *p < 0.05 and **p < 0.01.

**
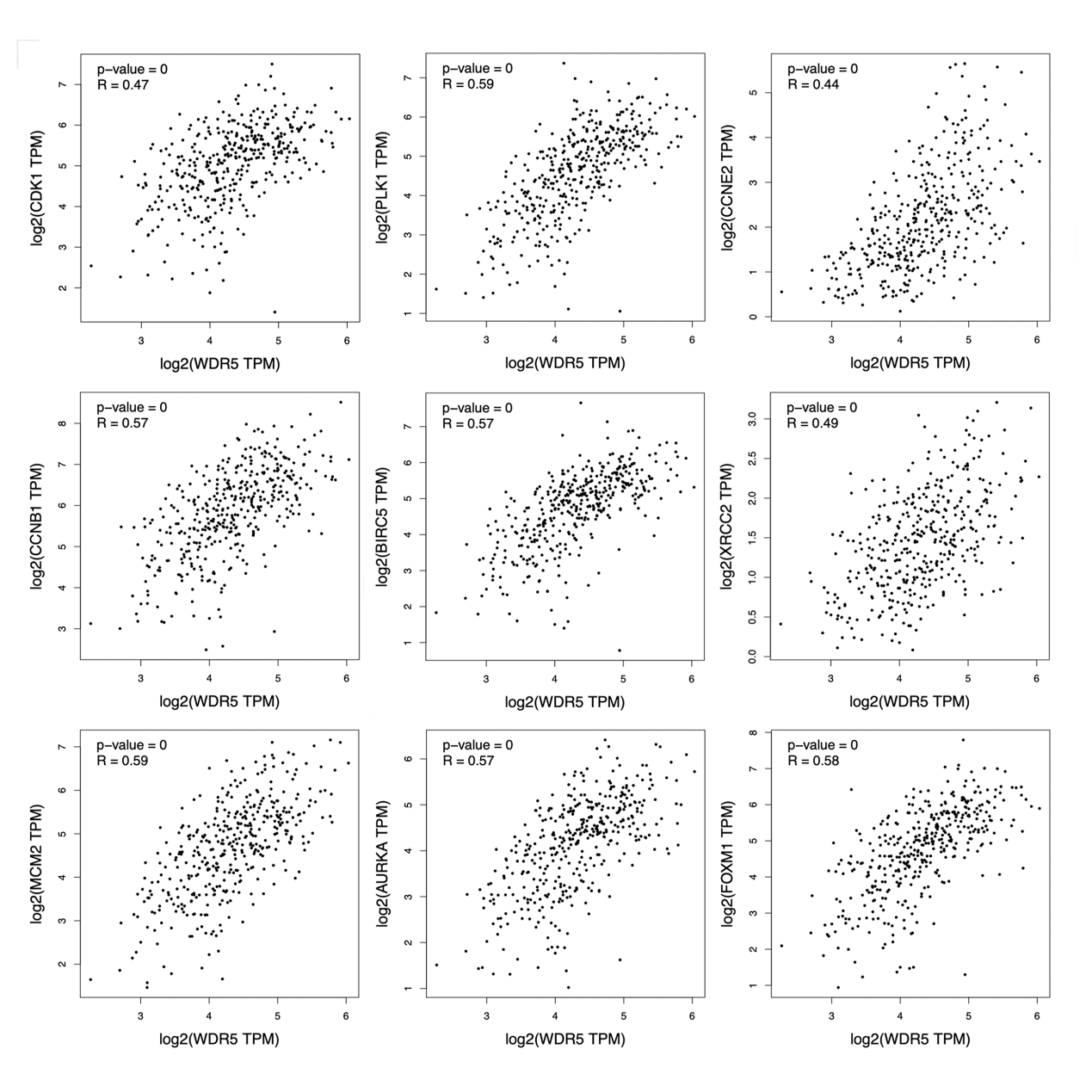
**

**Supplementary Figure 7. Pearson correlations between the expression of WDR5 and CDK1, PLK1, CCNE2, CCNB1, BIRC5, XRCC2, MCM2, AURKA and FOXM1 in TCGA cohort.**

**
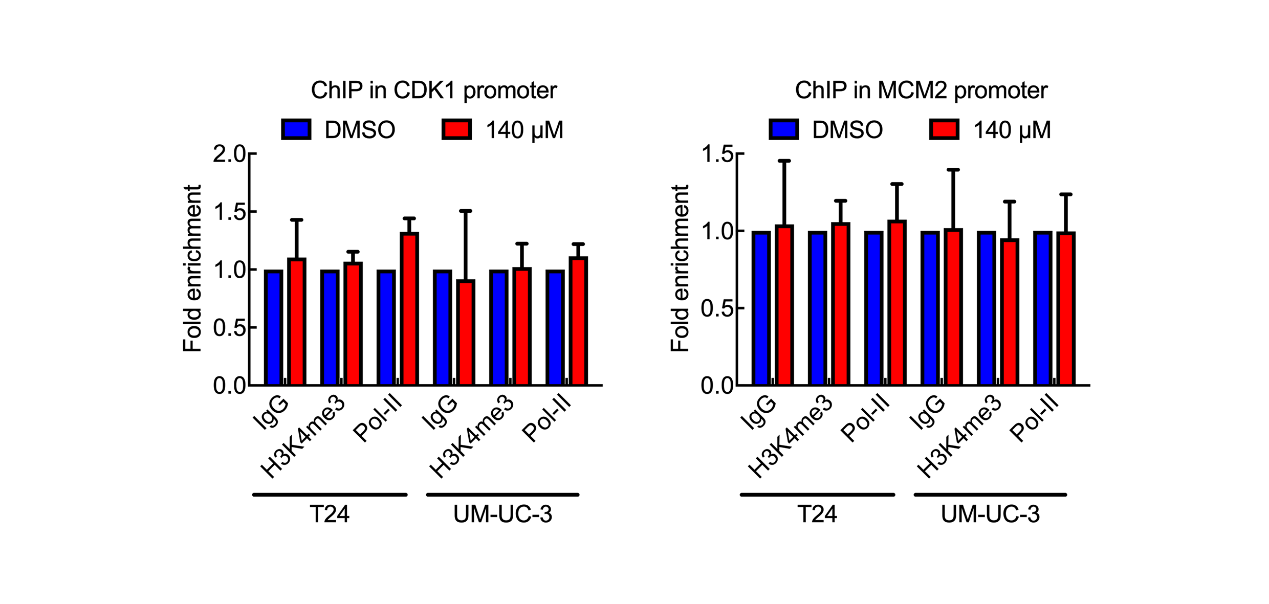
**

**Supplementary Figure 8. OICR-9429 treatment did not affect H3K4me3 and RNA polymerase-II levels on the promoter regions of CDK1 and MCM2 in UM-UC-3 and T24 cells.** *p < 0.05 and **p < 0.01.


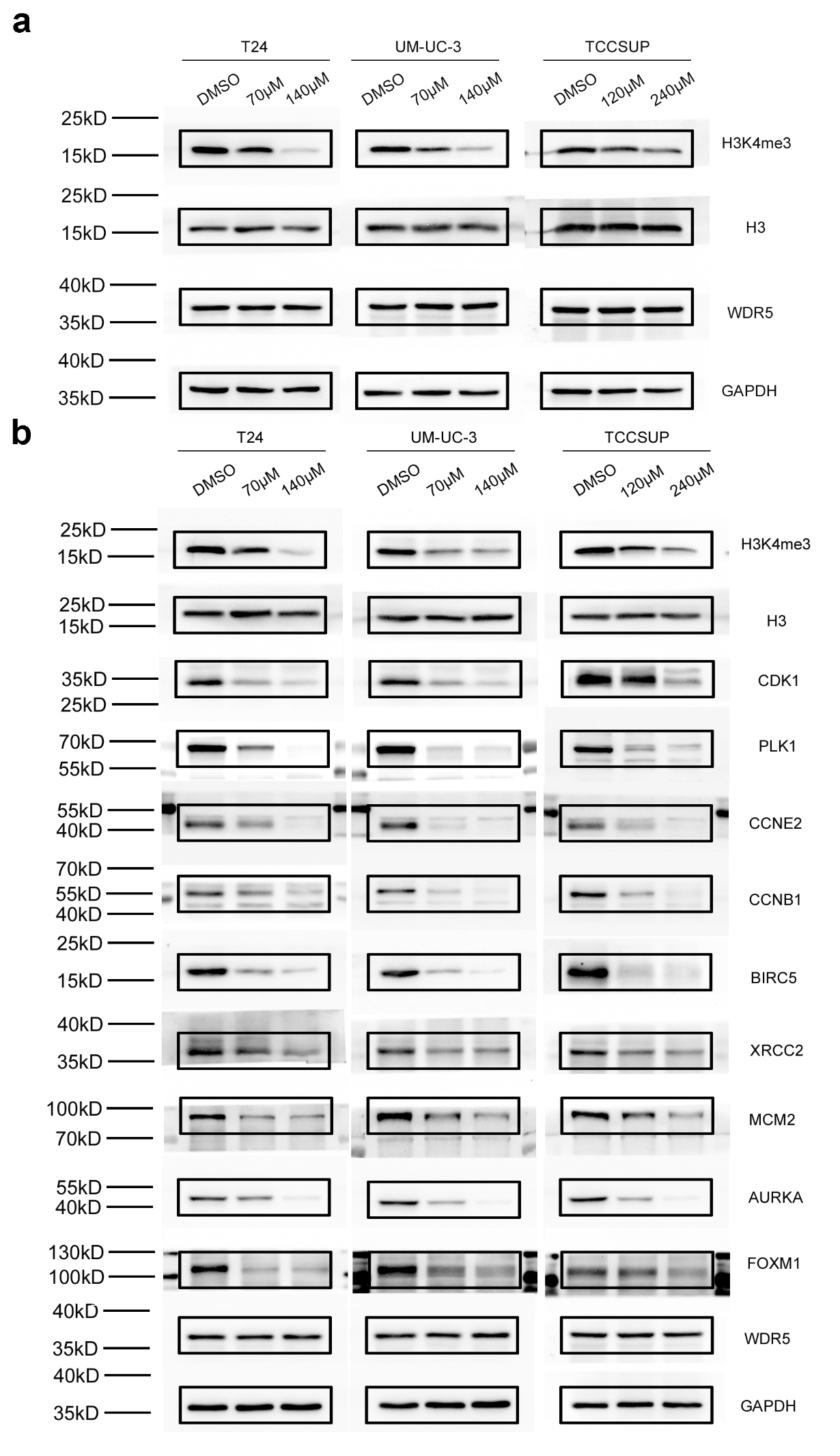


**Supplementary Figure 9. Original images of western blotting**

**a.** Original images of western blotting detection of H3K4me3, H3, WDR5 protein levels in OICR-9429 treating BCa cells in Figure 1g. **b.** Original images of western blotting detection of OICR-9429 targeted genes protein levels in three BCa cells in Figure 6d.

**Supplementary Table 1. Univariate and multivariate analysis of factors associated with overall survival in 345 cases of bladder cancer from TCGA cohort.**

|  | | Univariate | | |  | Multivariate | | |
| --- | --- | --- | --- | --- | --- | --- | --- | --- |
| Variable | | HR | 95% CI | *p*-Value |  | HR | 95% CI | *p*-Value |
| Gender (female/male) | | 1.163 | 0.823-1.643 | 0.392 |  |  |  | NA |
| Age, years (≥65/<65) | | 1.934 | 1.336-2.799 | **<0.001** |  | 2.033 | 1.401-2.949 | **<0.001** |
| Tumor stage (T3–T4/T1–T2) | | 1.895 | 1.277-2.811 | **0.002** |  | 1.543 | 1.028-2.317 | **0.036** |
| Nodal metastasis (N1–N2/N0) | | 2.269 | 1.652-3.116 | **<0.001** |  | 2.118 | 1.529-2.935 | **<0.001** |
| Histological grade (High/Low) | | 4.383 | 0.611-31.426 | 0.142 |  |  |  | NA |
| WDR5  (high/low) | | 1.385 | 1.005-1.907 | **0.046** |  | 1.478 | 1.071-2.040 | **0.018** |
|  | Univariate and multivariate analysis. Cox proportional hazards regression model. Variables associated with survival by univariate analyses were adopted as covariates in multivariate analyses. Significant *p*-values are shown in bold font. HR > 1, risk for death increased; HR < 1, risk for death reduced. | | | | | | | |

**Supplementary Table 2. Primers used in qPCR.**

| Primer Name | Sequence 5’-3’ |
| --- | --- |
| CDK1 Forward | GGAAACCAGGAAGCCTAGCATC |
| CDK1 Reverse | GGATGATTCAGTGCCATTTTGCC |
| PLK1 Forward | GGATGATTCAGTGCCATTTTGCC |
| PLK1 Reverse | ACCTTGGTGGAATGGTCAGGC |
| CCNE2 Forward | CGTTTACAAGCTAAGCAGCAG |
| CCNE2 Reverse | CCTGGGTAGTTTTCCTCTTC |
| CCNB1 Forward | TAAGGCGAAGATCAACATGG |
| CCNB1 Reverse | TTACCAATGTCCCCAAGAGC |
| CCNA2 Forward  CCNA2 Reverse  BIRC5 Forward  BIRC5 Reverse  XRCC2 Forward  XRCC2 Reverse  AURKA Forward  AURKA Reverse  E2F1 Forward  E2F1 Reverse  MCM2 Forward  MCM2 Reverse  FOXM1 Forward  FOXM1 Reverse  GAPDH Forward  GAPDH Reverse | CTCTACACAGTCACGGGACAAAG  CTGTGGTGCTTTGAGGTAGGTC  CCACTGAGAACGAGCCAGACTT  GTATTACAGGCGTAAGCCACCG  TCTGTTTGCTGATGAAGATTCACC  CATCGTGCTGTTAGGTGATAAAGC  CATCGTGCTGTTAGGTGATAAAGC  ATGGAGCATGTACTGACCACC  GGACCTGGAAACTGACCATCAG  CAGTGAGGTCTCATAGCGTGAC  TGCCAGCATTGCTCCTTCCATC  AAACTGCGACTTCGCTGTGCCA  TCTGCCAATGGCAAGGTCTCCT  CTGGATTCGGTCGTTTCTGCTG  GTCTCCTCTGACTTCAACAGCG  ACCACCCTGTTGCTGTAGCCAA |

**Supplementary Table 3. Primers used in ChIP-qPCR.**

| Primer Name | Sequence 5’-3’ |
| --- | --- |
| CDK1 Forward | GGGCAAAAGACATGAACAGACA |
| CDK1 Reverse  CCNE2 Forward  CCNE2 Reverse  CCNB1 Forward  CCNB1 Reverse  BIRC5 Forward  BIRC5 Reverse  XRCC2 Forward  XRCC2 Reverse  AURKA Forward  AURKA Reverse  MCM2 Forward  MCM2 Reverse  FOXM1 Forward  FOXM1 Reverse  PLK1 Forward  PLK1 Reverse | GGCATTCTGACTGGTTTGAGA  GGAAAATAGAAAGCAGAAAG  CTTATTGGCTTTAGGAGAAC  CGCCCTGGAAACGCATTCTCT  AGAAGAGCCAGCCTAGCCTCA  TGTTGGGATTACAGGCGTGAG  TGTGCCGGGAGTTGTAGTCCT  CTCCCACTGAACAAAATAATA  CTGCTAATTATGACACTTTAG  ACGGCTGAGCTCTTGGAAGAC  CTCGTCCGCCACTGAGATATC  CTCCTTTTGACTTGCTGTTG  CAAAAGCAAAACCCTAAACG  ACATCAGGCCTCAGGAAATCA  GAGGAGTCTGGGATGGGAAAA  GCCCCTACAACCACAGTGTAA  CACCAAGTTCCACTCACTCCT |
| Negtive-Forward | GTAATCAGGAAACTGCATAC |
| Negtive-Reverse | CTCAAGACTCAATAGTGATC |
